# The evolution of atmospheric particulate matter in an urban landscape since the Industrial Revolution

**DOI:** 10.1038/s41598-023-35679-3

**Published:** 2023-06-02

**Authors:** Ann L. Power, Richard K. Tennant, Alex G. Stewart, Christine Gosden, Annie T. Worsley, Richard Jones, John Love

**Affiliations:** 1 Biosciences, Faculty of Life and Health Sciences, University of Exeter, Exeter, UK; 2 Ex - Cheshire and Merseyside Public Health England Centre, Liverpool, UK; 3 Strata Environmental, 16 South Erradale, Gairloch, Scotland, UK; 4 Geography Department, University of Exeter, Exeter, UK

**Keywords:** Environmental sciences, Environmental impact

## Abstract

Atmospheric particulate matter (PM) causes 3.7 million annual deaths worldwide and potentially damages every organ in the body. The cancer-causing potential of fine particulates (PM_2.5_) highlights the inextricable link between air quality and human health. With over half of the world’s population living in cities, PM_2.5_ emissions are a major concern, however, our understanding of exposure to urban PM is restricted to relatively recent (post-1990) air quality monitoring programmes. To investigate how the composition and toxicity of PM has varied within an urban region, over timescales encompassing changing patterns of industrialisation and urbanisation, we reconstructed air pollution records spanning 200 years from the sediments of urban ponds in Merseyside (NW England), a heartland of urbanisation since the Industrial Revolution. These archives of urban environmental change across the region demonstrate a key shift in PM emissions from coarse carbonaceous ‘soot’ that peaked during the mid-twentieth century, to finer combustion-derived PM_2.5_ post-1980, mirroring changes in urban infrastructure. The evolution of urban pollution to a recent enhanced PM_2.5_ signal has important implications for understanding lifetime pollution exposures for urban populations over generational timescales.

## Introduction

Exposure to urban atmospheric particulate matter (PM) is a ‘silent killer’^1^, with acute and chronic health implications for every organ in the body^2^. Derived mainly from anthropogenic activities including power generation, domestic heating and transport, PM_10_ and PM_2.5_ (≤ 10 µm and ≤ 2.5 µm in aerodynamic diameter, respectively) are important urban health hazards^3,4^ and have been linked to serious diseases ranging from childhood asthma^5^*,* chronic obstructive pulmonary disease (COPD)^6^, heart disease^7^, stroke^8^, neurodegenerative disease^9^ to premature mortality^10^; they can affect vital organs including the brain^11,12^, with prenatal and early life exposure linked to autism^13^. Black carbon nanoparticles have been detected in the lung, liver and brain tissue of unborn babies, demonstrating a burden of pollution exposure even before birth^14^. Recent studies estimate that air pollution is responsible for more deaths world-wide than obesity and alcohol consumption combined, and is a comparable risk to tobacco smoking^15^. Although associations between PM_2.5_ levels and health have been made^16^, recently, the mechanistic triggers of PM_2.5_ in causing lung cancer have been discovered^17^ highlighting the inextricable links between human health and air quality, and the crucial need to understand urban population exposure to PM. Although urban PM is a global problem, understanding human exposure at the local level is crucial since urban PM is not geographically uniform in its composition^18^. Ambient PM varies from place to place^19^ and inevitably, over time, dependent on localised industrial activity, urbanisation, transport networks, fuel consumption and pollution control legislations. It is widely perceived that air quality in the UK has improved since the infamous London smog events during the 1950’s, due to the subsequent introduction of the Clean Air Act (1956) and increasingly stringent air quality legislations since this time. However, contemporary PM monitoring does not extend beyond the 1990’s and, therefore, we simply do not know how the nature of PM has changed over time with the evolution of the urban environment, changing fuel sources and pollution controls.

**Figure 1:**
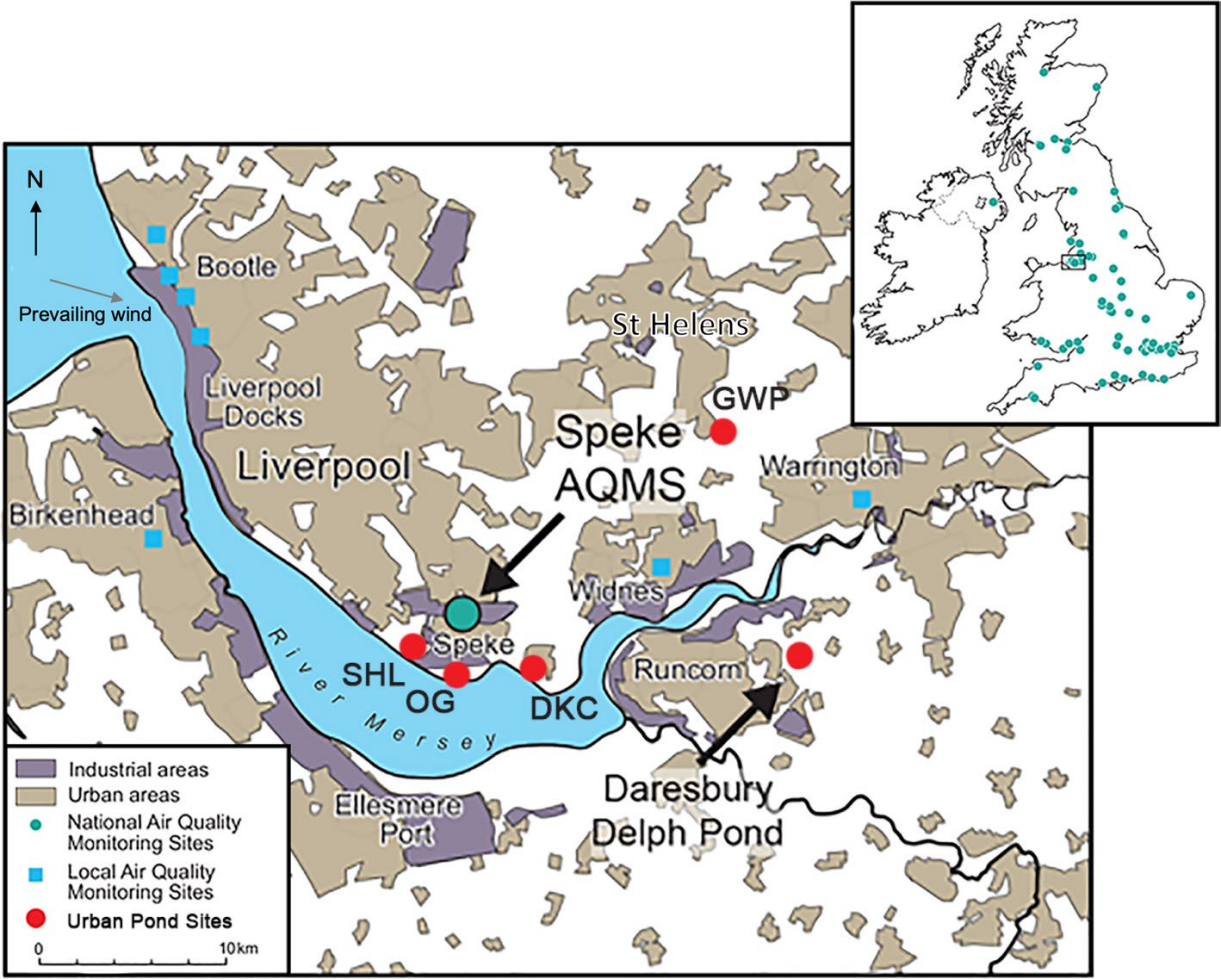
Distribution of air quality monitoring stations in the UK and Merseyside, and locality of investigated urban lakes and ponds. National air quality monitoring stations (AQMS) measuring PM_10_ and PM_2.5_ in the UK are shown as green circles. PM_10_ was analysed from the AQMS at Speke (National Grid Reference (NGR): SJ 43887, 83600) in Liverpool Unitary Authority (UA), which has been operational since 21-05-2003 and has been able to differentiate between PM_10_ and PM_2.5_ since 17-09-2008. Sediments used to analyse historic levels of PM were collected from Daresbury Delph Pond (NGR: SJ 57373, 81958), selected as a representative site for the region due to prevailing NWN wind direction; Dogs Kennel Clump (DKC) (NGR: SJ 46344, 82105) located in Halton UA; Speke Hall Lake (SHL) (NGR: SJ 41960, 82789) and Oglet (OG) (NGR: SJ 43491, 81845) located in Liverpool UA; and Griffin Wood Pond (GWP) (NGR: SJ 3705, 90962) located in St Helens UA (Table 1). Map (1:400,000 scale) contains OS data Crown copyright and database rights 2023 Ordnance Survey (100025252), accessed at http://digimap.edina.ac.uk.

PM_10_ and PM_2.5_ have been monitored since 1992 and 2009, respectively in the UK, by 77 air quality monitoring stations (AQMS) nationwide (Fig. 1) with typically only one or two AQMS used to determine air quality for an entire conurbation. Concentrations are recorded as bulk mass, but neither the source, size distribution, shape nor chemical composition of PM are determined^20^. Therefore, to understand long-term PM exposures and assess how air pollution has changed over time, we extend the urban PM pollution record to beyond conventional monitoring by reconstructing localised, long-term proxy air pollution histories (200 years) from the sediments of urban ponds within the Merseyside region, an industrial heartland of NW England (UK) (Fig. 1).

The Merseyside region has undergone a series of industrial and urban developments since the Industrial Revolution and remains a UK centre for chemicals production. Furthermore, populations in Merseyside suffer some of the worst life expectancy and health statistics in England^21^ (SI Table S1, Fig. S1) that cannot be accounted for by socio-economic factors alone; even when deprivation is taken into account, associations between mortality and living in close proximity to industry have been found^22^. The potential contribution of locality to disease, through current urban activities and industrial legacy, must therefore be considered when evaluating health inequalities, emphasising the need for higher spatially- and temporally-resolved air pollution data.

The aim of this study was to extend the record of air pollution monitoring beyond conventional PM_10_ data to assess long-term, generational changes in the nature of air pollution over time in a highly industrialised urban landscape. We used proxy records of pollution deposition from natural sediment archives, via geomagnetic, geochemical and spheroidal carbonaceous particulate (SCP) analyses. Several urban ponds throughout the Merseyside conurbation (Fig. 1) were investigated to reconstruct a cross-regional air pollution history. A 200-year history of air pollution deposition reconstructed from Daresbury Delph Pond, in Rucnorn is presented as an exemplar of the changing nature of pollution in the region influenced by urbanisation and industrialisation. A progressive increase in larger, carbonaceous particles derived from coal combustion that peaked during the mid-twentieth century, and a subsequent shift to finer PM_2.5_ emissions since the 1980s, is revealed by an increase in fine magnetic inorganic ash spheres (IAS). Evidence for a recent enhancement in relatively fine, combustion-derived air pollution is further supported by several other pond sites that exhibit localised signatures of fine magnetic PM post-1980. We also characterised urban PM_2.5_ archived on local AQMS filters from 2003 using scanning electron microscopy with energy dispersive spectroscopy (SEM–EDS) to determine the prevalence and potential sources of PM_2.5_ in the region which included combustion-derived, fine iron-rich spherules, as observed in the sediment records. We discuss the importance of these long-term proxy records of air pollution, which can inform critical insights into long-term releases of PM pollution within a conurbation, offering new possibilities for understanding how exposure to urban PM has changed over several generations. Such high-resolution (spatial and temporal) air pollution records reveal how pollution controls, changing industrial activities and increased air and road travel have altered the composition of urban PM over time, providing opportunities for a deeper understanding of links between air quality and human health, as well as assessing the efficacy of air quality legislations.

**Table 1:** Location and morphological data for investigated pond sites and core extraction dates.

Pond site	Core code	Core extraction date	Location (Easting, Northing)	Altitude (m)	Lake area (m^2^)	Lake-to-catchment ratio	Max. water depth (m)
Speke Hall Lake (SHL)	SHL1	08-09-2000	341960, 382789	23	3915	1:1874	3
	SHL2	19-09-2013					
Oglet Pond (OG)	OG	08-09-2000	343491, 381845	19	200	1:1.333	1.75
Dogs Kennel Clump (DKC)	DKC1	08-09-2000	346344, 382105	15	1700	1:1227	1.75
	DKC2	18-09-2013					
Griffin Wood Pond (GWP)	GWP	30-04-2014	353705, 390962	35	280	1:1.214	1
Daresbury Delph Pond (DDP)	Basins: ADD and BDD	09-03-2005	357366, 381958	55	770	1:1417	2

## Results and discussion

### Investigated urban sediment archives

We extended the record of PM pollution in Merseyside to before the start of air quality monitoring programmes using archives of atmospheric PM derived from lacustrine sediments, with sub-decadal resolution. To reconstruct histories of pollution deposition within an urban conurbation, we investigated small, human-made ponds, with restricted catchment areas^23^ defined by steep vegetated pond margins, located close to the highly industrialised towns of Runcorn and Widnes and the Speke AQMS (Fig. 1).

Daresbury Delph Pond (DDP) was selected as a site representative of the Merseyside region due to its longevity, extensive sediment record and location down-prevailing wind of industries at Ellesmere Port, Liverpool, Widnes and Runcorn (Fig. 1). Other pond sites investigated within the Merseyside conurbation include Speke Hall Lake (SHL), Dogs Kennel Clump (DKC), Oglet Pond (OG) and Griffin Wood Pond (GWP) (Table 1), providing supporting evidence of localised and regional pollution deposition.

**Figure 2:**
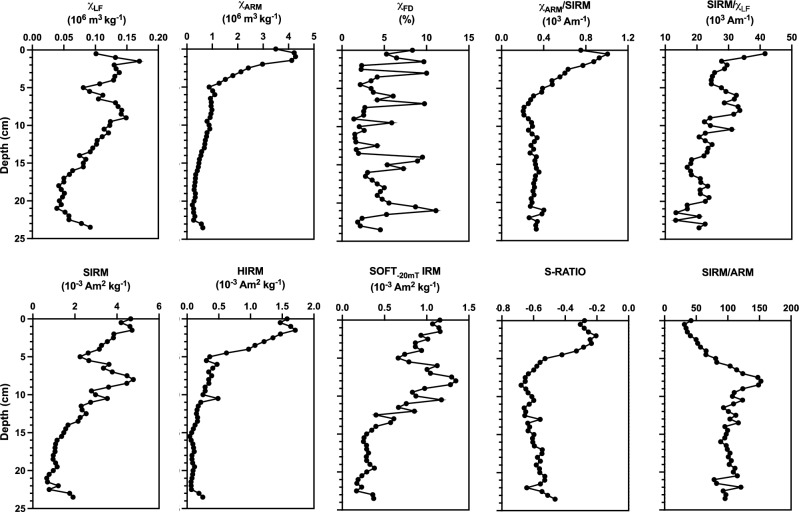
Geo-magnetic analysis of the sediment record from Daresbury Delph Pond. Down-core variations in magnetic concentration, mineralogy and grain size determined from concentrations of magnetic susceptibility (χ_LF_), susceptibility of anhysteretic remanent magnetism (χ_ARM_), susceptibility frequency dependence (χ_FD_), saturation isothermal remanence magnetisation (SIRM), hard isothermal remanence magnetization (HIRM) and inter-parametric ratios: S-RATIO (SIRM normalised to 100 mT backfield isothermal remanence magnetisation (IRM)), SIRM/χ_LF_, SIRM/ARM and χ_ARM_/SIRM.

Historic maps, documented management history and preliminary sedimentological analyses indicated that these pond sites had not been subjected to disturbance (e.g., dredging) or periodic drying, and exhibited minimal modification of their pond margins since their formation (SI Fig. S2). Intra-site reproducibility of geomagnetic profiles was determined by magnetic susceptibility (χ_LF_) (SI Figs. S3, S5, S6, S8) to further exclude sediment disturbance. Achievement of radiometric chronologies (SI Figs. S4–S8, SI Tables S2–S4) and extended geomagnetic analyses (Fig. 2) further confirmed the integrity of the sediment records and dominance of atmospheric-derived inputs. The air pollution archives reconstructed from each site provided local evidence of environmental change at different temporal scales (> 200 to 50 years) which, when combined, allowed assessment of cross-regional spatial variations in the history of PM deposition throughout Merseyside.

### A 200 year history of pollution deposition reconstructed from Daresbury Delph Pond

**Figure 3:**
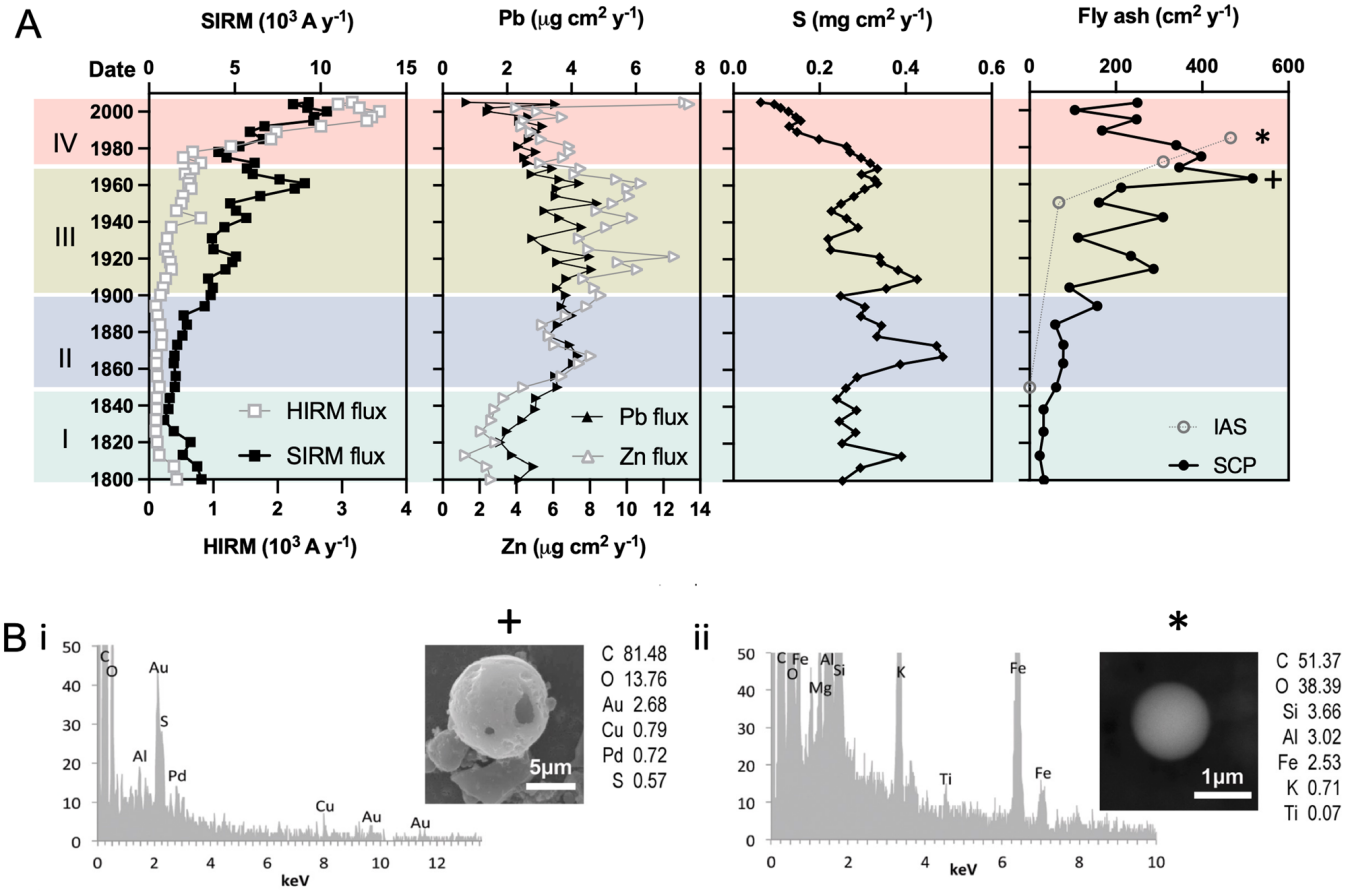
A history of air pollution deposition reconstructed from sediments of Daresbury Delph Pond (DDP), Runcorn (Halton). (**A**) Flux profiles for geomagnetic (SIRM, HIRM), geochemical (Pb, Zn, S) and fly ash particulate (spheroidal carbonaceous particles (SCP) and inorganic ash spheres (IAS)) pollution indicators. Concentration data were normalised for sedimentation accumulation rates to assess supply to the pond over time. (**B**) Exemplar pollution particles: SEM–EDS spectra, SEM images and corresponding mass of chemical elements (wt%) for representative SCP (i) and IAS (ii) extracted from the sediment horizons of 1963 (± 8 years) (**+**) and 1981 (± 6 years) (*) demonstrating the presence of fly ash within PM_10_ (i) and PM_2.5_ (ii) size fractions. Au and Pd peaks (i) are derived from the coating of the sample during preparation for SEM.

An air pollution history for Merseyside spanning 200 years (± 36 years) is presented from the sediments of DDP (Fig. 2) via geomagnetic characterisation (Fig. 2, Table 2); geomagnetic, geochemical and SCP flux profiles (Fig. 3A) and SEM analysis of pollution particles captured within the sediment record (Fig. 3B). Initially, the down-core geomagnetic properties of DDP were characterised to decipher the dominating magnetic signals within the sediment stratigraphy (Fig. 2). Magnetic grains in sediment archives can derive from the surrounding catchment (e.g., soil grains), in situ formation (e.g., bacterial magnetosomes, reduction diagenesis and post depositional changes) or direct atmospheric deposition (e.g., pollution particles), and their source can be deciphered by their concentration, size and mineralogy (SI Table S5).

**Table 2:** Explanation of magnetic parameters used to characterise down-core changes in the geomagnetic record of the urban pond sediments^24–26^.

Parameter	Interpretation
Low frequency susceptibility (χ_LF_)	Ferrimagnetic concentration. Low values may indicate the presence of paramagnetic minerals. The signal may be dominated by antiferromagnetic content if the sample has little or no ferrimagnetic component
Susceptibility frequency dependence (χ_FD_%)	High χ_FD_% reveals a superparamagnetic (SP) component and low χ_FD_% indicates a lack of SP grains
Anhysteretic remanence magnetisation (ARM) and susceptibility of ARM (χ_ARM_)	Sensitive to concentrations of fine-grained magnetic particles, particularly those of the stable single domain (SSD) grain size (0.03–0.5 µm)
Saturated isothermal remanence magnetisation (SIRM)	Reflects the concentration of all remanence-carrying minerals. SIRM is also sensitive to magnetic grain size and reflects the magnetic mineral assemblage
SOFT_−20mT_ IRM	Indicative of ‘soft’/multi domain (MD) ferrimagnetic grains
HIRM	Indicative of ‘hard’/antiferromagnetic content
S-RATIO	Indicative of soft and hard mineral contributions. S-RATIO values ~ < − 0.7 are ‘soft’ magnetic grains; > − 0.3: dominating antiferromagnetic component. Intermediate values ~ -0.4 to ~ 0.6: dominant SD ferrimagnetic signal with potential mix of antiferromagnetic grains
SIRM/χ_LF_	Low ratios: paramagnetic minerals; high ratios: canted-antiferromagnetic minerals. For samples with similar mineralogy, SIRM/χ_LF_ can also be used to assess grain size variations. High values: SSD grains; low values: SP/MD grains
χ_ARM_/SIRM	Discriminates variations in magnetic grain size. High ratios: SSD ferrimagnetic grains; low values: coarser (MD) grains
SIRM/ARM	Indicative of magnetic grain size variations in samples dominated by ferrimagnetic minerals. Low ratios: SSD grains; high ratios: MD grains

The χ_LF_ values of DDP sediments (0.039 to 0.17 10–6 m^3^ kg^−1^) highlight a potential mix of ferrimagnetic, diamagnetic, antiferromagnetic or paramagnetic minerals^24^, however, SIRM/χ_LF_ values (11.949 to 34.916 k Am^−1^) support a dominating magnetite signal and excludes significant contributions of authigenic iron sulphides (pyrite or greigite)^27^. A dominant catchment-derived input is excluded by χ_FD_ values < 8%, which indicates that superparamagnetic (< 0.02 μm) grains, typical of eroded soil particles, do not significantly contribute to the sediment record^28^ (Fig. 2). Positive correlations between magnetic concentration parameters (χ_LF_ and SIRM) and SCPs, unambiguous indicators of air pollution^29^, further confirms that an atmospheric pollution signal overrides any potential catchment-derived inputs (SI Table S6); highlights a coarse-grained (MD) magnetite signature as an overall indicator of air pollution; and supports the use of SIRM and χ_LF_ as proxies for total particulate pollution deposition^30–34^. As demonstrated in previous studies, urban PM contains magnetic grains produced by combustion and non-combustion processes from a range of industrial^31,35–39^, and transport activities^40,41^. Sources of anthropogenic magnetic grains can be traced via their magnetic properties (SI Table S5).

A distinct shift to a relatively fine grained and antiferromagnetic component occurs in the upper section of the DDP core (0–5 cm) as indicated by proportionately higher increases in χ_ARM_ compared to χ_LF_, an increase in SIRM/χ_LF_ values and a shift in S-RATIO (to >  − 0.3). DDP demonstrates a steadily increasing trend in HIRM throughout the twentieth century, with a further enhancement in recent (post-1985 (± 5 years) sediment. As well as coarse magnetite grains, transport and industry also generate magnetite nanoparticles^9,42^ and fine (< 3 μm) iron spherules^31,35–39,43^. Inorganic ash spheres (IAS), produced from fossil fuel combustion are typically comprised of aluminium silicates with varying amounts of iron and are rich in both haematite and magnetite^34,44^; therefore, HIRM, indicative of haematite contribution, is used as a proxy for fly ash deposition^33^.

To assess the supply of PM to DDP over time, down core SIRM and HIRM flux profiles (concentration data normalised for sediment accumulation rates achieved from ^210^Pb dating (SI Table S2)) are plotted alongside other typical anthropogenic indicators including Pb, S, Zn, SCPs and IAS (Fig. 3A). Long-term changes in pollution characteristics over generational timescales can therefore be determined (SI Tables S7, S8). Four main temporal phases of pollution deposition were identified, determined by the geomagnetic properties of DDP. Key changes in the pollution flux profiles correspond to patterns of regional industrial activity, urbanisation, transportation infrastructure and pollution regulation since 1800 (Fig. 3A):

- *Phase I* Pre-1850 (± 28 years). Low scale, localised industrial activity at this time is reflected by relatively low pollution levels.

Runcorn was emerging as a prominent port at the start of the nineteenth century. Improved transport links (canals and railway) resulted in a rise in quarrying and shipbuilding^45^. The first early ‘Leblanc’ chemical works in Runcorn was established from 1803^46^, with chemical industries expanding to Widnes from 1847^47^.

- *Phase II* 1850 (± 28 years) to 1900 (± 20 years). Expansion and intensification of Leblanc chemical industries throughout Merseyside is marked by an enhancement in Pb and S pollution.

Leblanc chemical production dominated industry in the region at this time^47^. A boom in the chemical trade from 1861 to 1875 resulted in increased production at existing factories and the construction of new sites^48^. In Widnes alone, 22 Leblanc chemical sites were established between 1847 and 1884^47^. The Leblanc process involved heating salt with sulphuric acid in lead chambers to produce saltcake, which in turn was heated with coal and limestone to produce black ash, from which alkali was recovered^49^. This may explain the distinct enhancement in Pb and S flux at this time.

- *Phase III* 1900 (± 20 years) to 1970 (± 8 years). Diversification and intensification of industry due to technological advancements and rapid urbanisation corresponds with elevated pollution levels (SIRM, Pb, S and Zn and SCPs) which peak during the mid-twentieth century.

Leblanc methods were replaced by modern techniques at the start of the twentieth century (e.g., Slovay and electrolytic chemical production)^50,51^ and the chemical industry diversified to include the production of organic chemicals, which led to the closure of tanneries in the 1960’s^45^. Polymers, plastics, polishes, perfumes, soaps, detergents, metal alloys, chlorine and chlorinated solvents, pharmaceuticals and agrochemicals were manufactured in the region^45,52,53^. Petrochemicals and oil refineries established at Ellesmere Port from 1920. The enhancement in Zn flux post-1900, highlights relatively modern (twentieth century) pollution sources^54,55^ e.g., the local manufacturing of car paint containing Zn additives^56^.

The observed increasing trend in pollution deposition throughout Merseyside from 1900 to 1970 also encompasses the demands of two world wars on industry. Significant urban expansion occurred post-WWII with industrial and residential developments across the region between 1950 and 1970 (e.g., at Runcorn, Halewood and Speke)^57,58^, resulting in population increases. Coal fired power stations opened at Ince (240 MW capacity, operational 1957 to 1997) and Bold (300 MW capacity, operational 1958 to 1991)^59^.

- *Phase IV* Post-1970 (± 8 years). The decline of SCPs, Pb, Zn and S reflects increasingly stringent air quality controls, however increases in SIRM, HIRM and IAS highlight a relatively fine combustion signal.

Steady declines in SCPs, S and Pb post-1970 reflect a time of increasingly stringent air quality legislation following the Clean Air Act of 1956 (e.g., Control of Pollution Act 1975, Motor Fuel Regulation 1981, Environmental Protection Act 1990 and United Kingdom Air Quality Strategy 1997)^60^. Sulphur and heavy metals were reduced in fuels and unleaded petrol was introduced in 1988 (EC Directive 85/210/EEC). Although declines in SCPs, Pb, Zn and S occur, this most recent temporal phase is characterised by a secondary pollution (SIRM and HIRM) peak and a distinct shift to a finer pollution signal as indicted by χ_ARM_ (Fig. 2), confirmed by the accumulation of magnetic IAS which are typical of high temperature combustion (Fig. 3A)^33,61^. SCP and IAS isolated from 1963 (± 8 years) and 1981 (± 6 years) sediment, respectively, show the composition and morphology of individual fly ash particles present in these sediments (Fig. 3B).

Fiddler’s Ferry Power station (2000 MW capacity), a potential key source of IAS opened in 1971 (operating until 2020)^62^. A wide range of chemicals continued to be produced in the region, with a major petrochemicals company manufacturing chlorine and fluorine-based products established in Weston during the 1990’s^63^. Continued urbanisation and increases in road and air travel are also likely to contribute to the pollution signal at DDP. This includes the expansion of Speke Airport, a small airbase opened in the 1930's, to John Lennon International Airport a major transport hub of the NW England; and the opening of the M56 motorway (700 m from DDP) and A56 dual carriageway which occurred between 1971 and 1975.

### Cross-regional evidence for a shift to fine PM_2.5_ pollution post-1980

**Figure 4:**
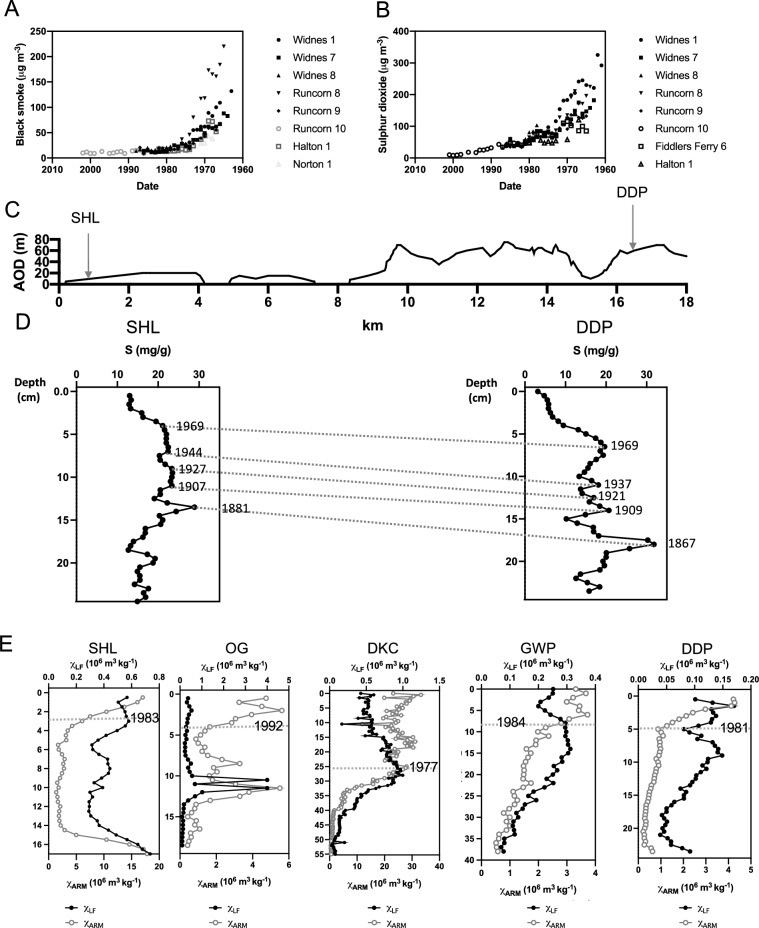
A cross-regional post-1800 air pollution signal for Merseyside reconstructed from urban ponds showing regional trends in sulphur deposition and a temporal shift to fine magnetic grains in recent sediments. (**A**) Mean annual black smoke and (**B**) sulphur dioxide concentrations monitored in Halton post-1961. Reproduced from the UK National Air Quality Archives (DEFRA) http://airquality.co.uk. Data were collated from several monitoring sites across the borough: Widnes 1 (NGR: SJ 513,854: 1963–1976), Widnes 7 (NGR: SJ 485,859: 1964–1986), Widens 8 (NGR: SJ 513,854: 1980–1989), Runcorn 8 (NGR: SJ 5108,31: 1965–1982), Runcorn 9 (NGR: SJ 519,821: 1966–1987), Runcorn 10 (NGR: SJ 511,833: 1984–2002) Halton 1 (NGR: SJ 536,819: 1967–1982) and Norton 1 (NGR: SJ 554,815: 1968–1986). (**C**) Topography of Merseyside from Daresbury (NGR: SJ 359, 382) to Speke (NGR: SJ 341, 382) showing the location of DDP in the east, and SHL in the west, of the region. (**D**) Post-1800 sulphur (S) concentrations recorded in sediments of SHL (core SHL1) and DDP (core BDD1) highlighting corresponding trends from the late nineteenth century: SHL peak at 1881 (± 24 years) and DDP peak at 1867 (± 26 years). Synchronicity in the ^210^Pb dates of S peaks between the two ponds highlights a likely regional atmospheric pollution signal. Declines in S deposition post-1970 (± 3 years at SHL and ± 8 years at DDP) mirror the decline in black smoke (**A**) and sulphur dioxide (**B**) concentrations monitored in Halton with significant statistical correlations observed (SI Table S9). (**E**) Concentration of total ferrimagentic grains (χ_LF_) and fine (stable domain) ferrimagentic grains (χ_ARM_) in post-1900 sediment from Daresbury Delph Pond (DDP: 1900 (± 20 years) to 2006 (± 0 years)), Speke Hall Lake (SHL1: 1900 (± 17 years) to 2001 (± 0 years)), Griffin Wood Pond (GWP:  ~ 1900 (± 11 years) to 2013 (± 0 years)), Dogs Kennel Clump (DKC1: DKC2:  ~ 1900 (± 23 years) to 2013 (± 0 years)) and Oglet Pond (OG:  ~ 1914 (± 21 years) to 2001 (± 0 years)). χ_LF_ trends are a proxy for overall particulate pollution deposition. Proportionately higher χ_ARM_ enhancements in the most recent sections of the cores indicate an increased contribution of relatively finer (~ < 2 μm) magnetic grains. All sites demonstrate an increase in the deposition of magnetic fines in recent (~ post-1980) sediments, highlighting a regional trend.

Several other urban pond stratigraphies from within the Merseyside region support the air pollution history obtained from DDP. Each pond site provides a unique, localised history of environmental change, which can be used collectively to understand spatial differences in particulate deposition within the region, over time (Fig. 4).

DDP and SHL, although located ~ 16.5 km apart (Fig. 4C) demonstrate a synchronised post-1800 cross-regional sulphur signal, determined by closely matched ^210^Pb dating of sulphur peaks (Fig. 4D). This is likely to reflect an atmospheric-derived regional depositional history of sulphur from coal combustion. Statistically significant correlations are observed between post-1960 sulphur concentrations from SHL and DDP and available post-1960 black smoke and sulphur dioxide data (Fig. 4A,B) monitored as part of the ‘National Survey’ in Halton (SI Table S9). Post-1969 declines in sulphur deposition exhibited by the ponds coincide with the introduction of the Clean Air Act (1956) that reduced domestic coal combustion. However, a distinct feature of the cross regional geomagnetic record is a post-1980 enhancement of fine (single domain) magnetic grains, typically ~  < 2 μm^64^, indicated by proportionately higher χ_ARM_ to χ_LF_ values exhibited in the recent sediments of SHL (post-1983 ± 2 years), OG (post-1992 ± 2 years), DKC (post-1977 ± 4 years) and GWP (post-1984 ± 3 years), as observed at DDP (Fig. 4E).

**Figure 5:**
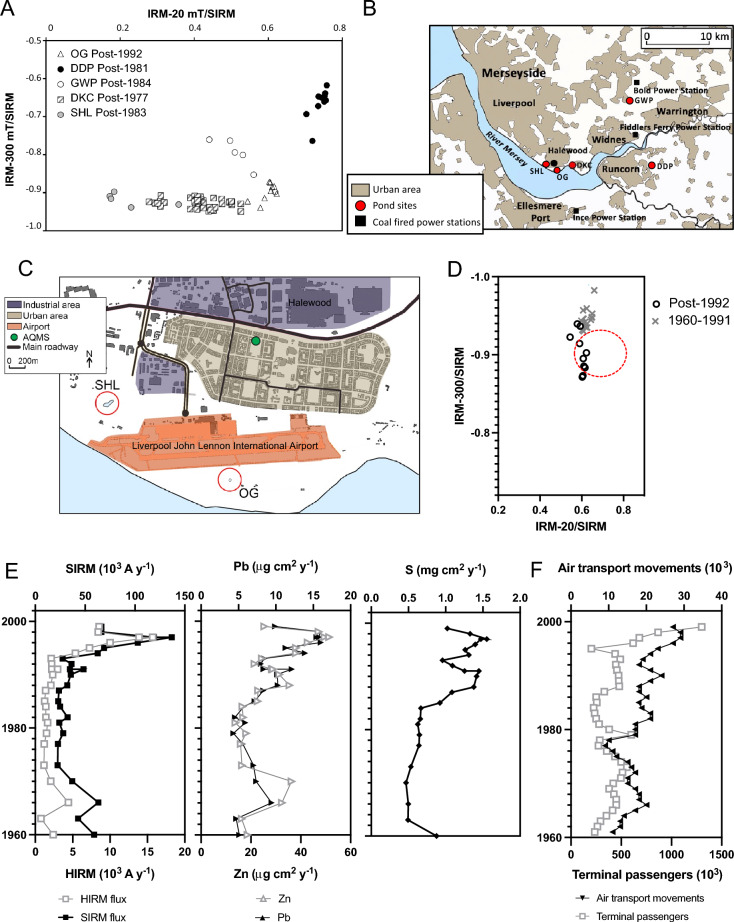
Distinguishing sources of pollution in recent urban pond sediments. (**A**) Discrimination of anthropogenic magnetic grains in recent sediments from Speke Hall Lake (SHL), Dogs Kennel Clump pond (DKC), Oglet pond (OG), Griffin Wood pond (GWP) and Daresbury Delph pond (DDP) using backfield isothermal remanent ratios (IRM-20 mT/SIRM and IRM-300 mT/SIRM), a variation of Hunt et al. (1984)’s magnetic bi-plot used to distinguish between fly ash and vehicular dusts^65^. Daresbury Delph Pond (DDP) and Griffin Wood Pond (GW) samples demonstrate a relatively ‘harder’ antiferromagnetic signal, characteristic of fly ash emissions. SHL and DKC display a ferrimagnetic dominated signal, typical of urban particulates, from traffic and industrial emissions, that are characteristic of a mix of coarse (multi domain), fine (stable domain) and ultrafine magnetic grains. OG exhibits a relatively finer ferrimagnetic signal in post-1990 sediments, indicated by a decrease in grain size observed with increasing IRM-20mT/SIRM values. (**B**) Locality of urban ponds to coal fired power stations in the Merseyside region: Ince (1957–1997); Bold (1958–1991) and Fiddlers Ferry (1973–2020). Map (1:400,000 scale) contains OS data Crown copyright and database rights 2023 Ordnance Survey (100025252), accessed at digimap.edina.ac.uk. (**C**) Proximity (< 300 m) of OG pond to the runway at Liverpool John Lennon International Airport. Map (1:25,000 scale) contains OS data Crown copyright and database rights 2023 Ordnancy Survey (100025252), accessed at digimap.edina.ac.uk. (**D**) Detection of aviation-derived magnetic grains, specifically from aircraft engines, in post-1995 OG pond sediments. Distinguished by IRM-20 mT/SIRM versus IRM-300 mT/SIRM measurements, post-1995 sediments overlap magnetic values (red dashed line) reported for aircraft engine particulates^33^. (**E**) Geochemical (Pb, Zn, S), and magnetic (SIRM, HIRM) flux profiles for OG pond. (**F**) Post-1960 air transport movements and terminal passengers recorded at John Lennon International Airport collated from Historical Annual Airport Tables produced by the Civil Aviation Authority http://www.caa.co.uk.

Further geomagnetic characteisation^65^ was used to determine potential sources of fine magnetic grains throughout the Merseyside region (Fig. 5A). The recent sediments of DDP and GWP exhibit relatively antiferromagnetic signals, characteristic of fly ash emissions, further supporting the observed IAS increase detected in DDP (Fig. 3A). The magnetic characteristics of GWP overlap those of fly ash particulates, indicating a potentially dominant pollution source from power stations^65^, which may include Bold Power station (2.5 km north of GWP) operational from the mid-1950’s to 1991; and Fiddlers Ferry Power station (4.6 km south of GWP), which opened in 1971 (Fig. 5B).

SHL and DKC located in the west of the region, exhibit a ferrimagnetic signal in their recent sediments, which is likely to suggest a mixed-source urban pollution signal, potentially reflecting particulates derived from a range of surrounding industries and transportation sources. We have previously reported for DKC notable, post-1950 increases in SIRM, SCP, Pb, Zn and Cu deposition which coincide with the establishment of foundries, chemical and pharmaceutical industries, car manufacturing and urban development at Halewood (< 3 km northwest of DKC) with continued localised urban development post-1980, and enhanced road and air travel, including the expansion of John Lennon International airport^23^. Ferrimagnetic grains 0–3 µm found in roadside dusts attributed to vehicular exhaust emissions^27^ are also likely to contribute to the urban pollution signal.

Located < 300 m from the runway at John Lennon International Airport (Fig. 5 C), OG exhibits a distinct shift in the magnetic signal post-1995 (Fig. 5A) that mirrors magnetic characteristics previously reported for combustion particles from aircraft engines^41^ (Fig. 5D). Furthermore, statistically significant correlations (SI Table S10) determined between post-1960 pollution flux profiles reconstructed from OG sediments (Fig. 5E) and available air travel statistics (Fig. 5F) further supports a fine-grained, localised pollution signal containing Pb and Zn.

### Characterisation of ambient urban PM_2.5_ in Merseyside

**Figure 6:**
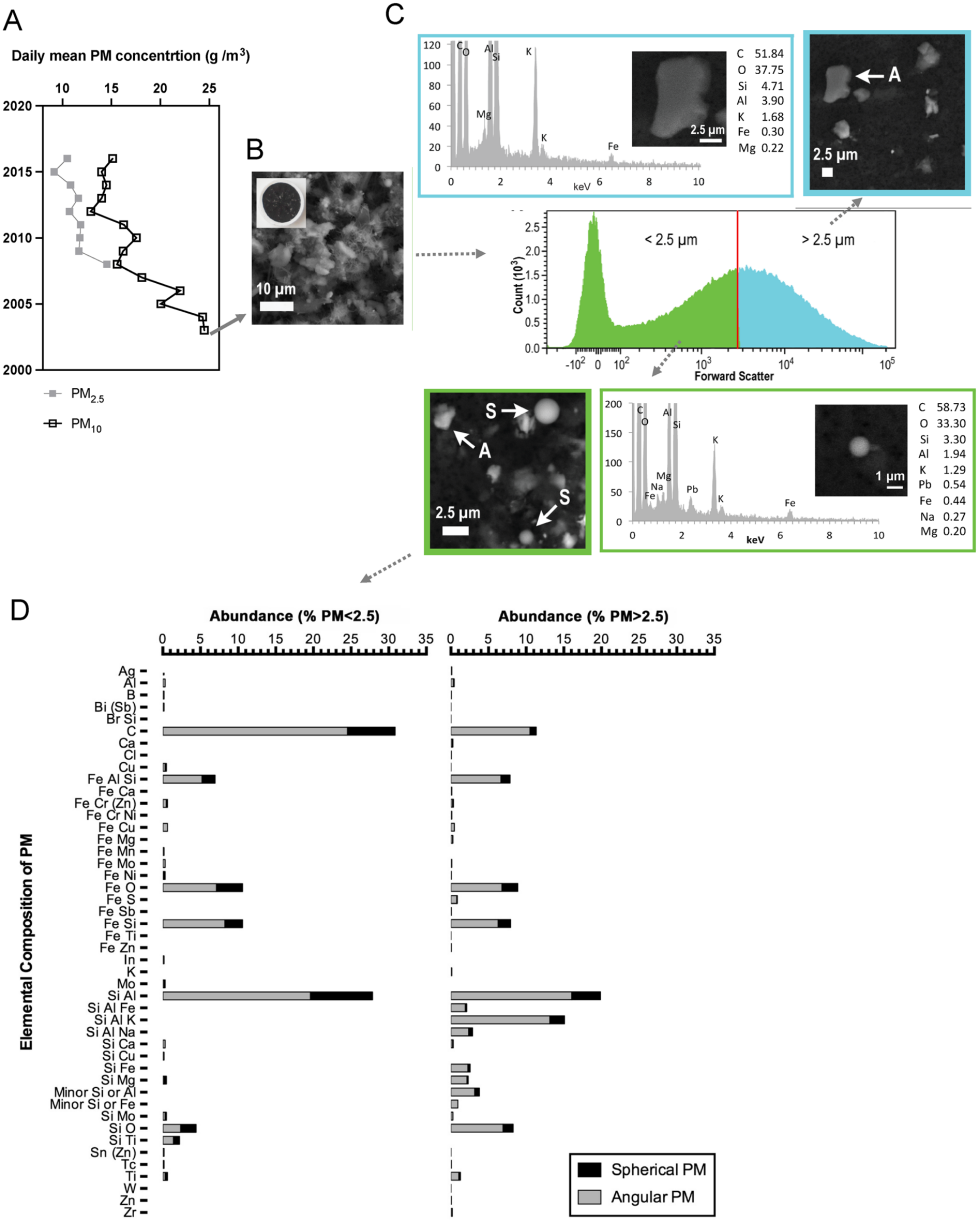
Characterisation of urban PM archived at Speke, Liverpool air quality monitoring station. (**A**) Daily mean PM_10_ and PM_2.5_ concentrations recorded at Speke, Liverpool air quality monitoring station, from commencement of monitoring (2003) to 2016. (**B**) Archived PM_10_ Tapered Element Oscillating Microbalance (TEOM) filter that collected PM_10_ from 08/09/2003 to 06/10/2003, prior to the AQMS’s capability to segregate between PM_10_ and PM_2.5_ with SEM images of the TEOM filter. (**C**) Extraction and analysis of PM_2.5_ and PM_10_ was achieved using flow cytometry and SEM–EDS. A histogram of the size distribution of PM removed from the TEOM filter was determined by the forward scatter properties of particulates, which were sorted via flow cytometry into PM < 2.5 µm (green) and PM 2.5 µm to 15 µm (blue). Exemplar angular (A) and spherical (S) PM are presented. SEM energy dispersive spectrometry (SEM–EDS) spectra, SEM images and corresponding mass of chemical elements (wt%) for selected particles are shown. Background elemental contributions from the filter are detailed in SI Table S11. (**D**) Relative abundance, composition and shape of PM < 2.5 µm. The geometric and chemical characteristics of 3,679 particulates were individually analysed by automated SEM–EDS. Particles were grouped into those with equivalent circular diameters < 2.5 μm (PM < 2.5) and > 2.5 μm (PM > 2.5) and classified by chemical composition (SI Table S12). Elements, including S and Pb, that were detected in < 1% of the PM are not shown here. Angular particulates are indicated by grey bars; spherical particulates are indicated by black bars.

To further explore the prevalence of PM_2.5_ in Merseyside, as observed in the recent sediments of the urban pond archives, we characterised the fine fraction of PM_10_ dust collected from a Tapered Element Oscillating Microbalance (TEOM) filter (Fig. 6B) sampled by the AQMS in Speke, Liverpool (Fig. 1) during 08/09/2003 to 06/10/2003 (Fig. 6A). Flow cytometry was applied to physically separate and concentrate PM_2.5_ from the bulk PM_10_ particulate (Fig. 6C) and individual particulates within this fine fraction were characterised by automated SEM analysis (Fig. 6D, SI Table S7).

PM with equivalent circular diameters (ECD) > 2.5 μm (PM > 2.5) (Fig. 6D) were detected, mainly (84.3%) angular particles composed primarily (71.9%) of silica with varying amounts of Al, K, Na, Fe, Ca and Ti, indicative of wind-blown soils, building erosion and crustal material^66,67^. Approximately 22% of angular PM > 2.5 contained Fe, S and Si and were likely derived from various natural and anthropogenic sources, notably soils, road dusts, street furniture, vehicle wear and potentially, nearby metallurgical works or metal recycling facilities^68–70^.

Approximately 57% of the concentrated fine particulates had an ECD < 2.5 µm (PM < 2.5); 70% of which were irregularly shaped, angular particles. Of these particulates, 22% were ferrous and contained Al, Si, S, Cu and Cr; 25% were carbonaceous and 19.6% were composed of aluminium silicate. The detection of Cu, Cr, Mo, Pb, Ni, Sn and Ag within the angular PM < 2.5 reflects the varied composition of urban PM, potentially from a variety of sources such as steel, paints, asphalt, vehicle abrasion and industrial dusts^71–73^.

Spherical particulates comprised approximately 27% of PM < 2.5. Spherules are characteristic of anthropogenic high-temperature combustion and industrial processes^40,66,74^. Approximately 25% of spheres contained only C. The remaining 75% of spheres primarily contained Al, Si and/or Fe, characteristic of IAS particles^44,68^, resembling the IAS observed in the recent sediments of DDP (Fig. 3Bii).

Direct comparisons of PM_2.5_ concentrations recorded by the AQMS at Speke was prevented by the restricted timeframe (post-1990) of monitoring, however, fine spheres detected in the PM_2.5_ fraction, mirrors the IAS detected in the sediments of DDP, further supporting the geo-forensic potential of urban ponds as archives of PM_2.5_ deposition in Merseyside beyond spatially and temporally restricted conventional monitoring. The detailed characterisation of PM_2.5_ via SEM–EDS from the TEOM filter demonstrates the complexity of urban particulate size, source and composition, highlighting the oversimplification of bulk mass measurements of ambient urban PM as is currently performed by the AQMS.

### Importance of long-term proxy records of PM_2.5_ pollution

The urban spatio-temporal records we have constructed in the Merseyside region allow a long-term (200 year) assessment of urban environmental change that spans the Great Acceleration (post-1950), a period of unprecedented urban development at regional, national and global scales. Pollution signals captured by the urban ponds demonstrate the heterogeneity of PM deposition within an urban landscape and over time, uncovering important changes in the nature of air pollution that is impossible to detect from temporally restricted conventional PM monitoring stations.

These records reveal the baseline characteristics of urban PM prior to PM_10_ and PM_2.5_ monitoring, showing how urban PM has changed over time in relation to known industrial and urban activity in the Merseyside region. Most notably, our reconstructions reveal a recent pollution peak after 1980 characterised by a rapid shift to fine combustion-derived PM_2.5_ that mirrors industry, road and air travel signals^27,30,31,35–39,41^.

It is widely assumed that urban air quality in the UK has improved since the visible smogs of the 1950’s however, we demonstrate a shift to more insidious and ‘invisible’ PM, despite the implementation of increasingly stringent air quality legislation since the introduction of the Clean Air Acts in 1956. It is possible that, as an archetypical and diversified urban/industrial landscape, the modern change to finer PM noted in Merseyside may be reflected nationally and internationally.

Indicative of a shift to enhanced releases of PM_2.5_ from combustion-derived sources in general, our work demonstrates the presence of a potentially more toxic urban pollution signature post-1980 since higher associations between mortality and long-term exposure to PM_2.5_, compared to PM_10_ have been reported, with no safe threshold below which PM_2.5_ does not effect health^75^. This has considerable implications for human health since fine particulates can penetrate deep into the lung and potentially translocate into the circulatory system, reaching the heart and cumulate in and damage other vital organs^11,76–78^. For example, magnetite nanospheres characteristic of urban high-temperature combustion particulates have been detected in human brain^9^ and heart tissue^79^. In addition, PM_2.5_ are carriers for toxic heavy metals such as Pb, Zn and Cd^31,35–39^. Recently, the way PM_2.5_ promotes cancerous changes in lung cells has been determined^17^ emphasizing the significance of PM_2.5_ exposure, which is largely unavoidable, on public health.

For the first time, we show that in Merseyside, urban generations born post-1980 have been exposed to higher levels of PM_2.5_ during their lifetimes compared to previous generations. Understanding the health effects of life-time, cumulative exposures to PM_2.5_ (and PM_10_) on chronic or long-latency diseases such as cancer is crucial for urban populations residing in complex industrialised regions that have experienced intense urban development. The proxy records of atmospheric PM deposition reconstructed from the urban ponds may be used to compliment socioeconomic and lifestyle information for the Merseyside population to help explain links between life-time exposures to PM and the prevalence of specific diseases experienced in Merseyside today.

## Conclusion

It is widely assumed that urban air quality in the UK has improved since the visible smogs of the 1950’s and the implementation of increasingly stringent national air quality legislations since the introduction of the Clean Air Act (1956). However, our reconstructions of urban air pollution in Merseyside using small human-made ponds, has allowed an assessment of how air pollution has changed during the intense urbanisation experienced in an industrial landscape since the 1800s, which is impossible to achieve with contemporary (post-1990) air quality monitoring. A key feature of the cross-regional signal is a distinct peak in pollution post-1980, characterised by a rapid shift to fine, combustion-derived PM. Geomagnetic characteristics indicate these PM derive from a number of urban sources including road transport, industrial activities and air travel. For the first time, we show that in Merseyside, urban generations born post-1990 have been exposed to higher levels of fine PM during their lifetimes compared to previous generations. These unique records offer new opportunities to understand PM exposure for urban populations, to help piece together the array of environmental factors to which urban populations are exposed to PM throughout their lifetimes, to ensure long-term help protect the health of urban populations in the future.

## Materials and methods

Sediment cores from five urban pond sites throughout Merseyside were analysed. Down-core geomagnetic profiles were used to initially assess sediment disturbance, and to determine if a signal of atmospheric deposition had recorded. Radiometric (^210^Pb) dating was used to assign reliable chronologies to the cores and calculate sediment accumulation rates. Anthropogenic deposition signals were further explored using geochemical analyses of key anthropogenic elements including lead (Pb), zinc (Zn) and sulphur (S), and the detection of pollution particulates: spheroidal carbonaceous particulates (SCPs) and inorganic ash spheres (IAS) via light microscopy. The size and elemental composition of individual SCPs and IAS particulate in the sediment archives were further analyzed using scanning electron microscopy with energy dispersive X-ray spectroscopy (SEM–EDS). The prevalence of IAS in more contemporary (2003) ambient atmospheric samples was also investigated by the analysis of PM_2.5_ collected on an air filter archived by an air quality monitoring station in the region. The size, shape and composition of individual particulates were analysed using automated SEM–EDS.

### Core extraction and pond catchment descriptions

Details of sediment cores collected for all investigated pond sites are provided (Table 1). Retrieved from the centre of each pond/lake basin using a hand-held gravity corer (Gilson), cores samples were stored at 5 °C, extruded at 0.5 cm intervals and dried at 35 °C.

### Geomagnetism

Magnetic susceptibility was measured at low (0.46 kHz) and high (4.6 kHz) frequencies using a Bartington MS2B sensor and MS2 meter. Ferrimagnetic concentration was determined by low frequency susceptibility normalised for sample weight (χ_LF_). Susceptibility frequency dependence (χ_FD_) was calculated as the percentage difference between high and low magnetic susceptibility frequencies to determine the contribution of superparamagnetic grains (< 0.05 μm)^24^.

A Molspin AF demagnetiser imparted an anhysteretic remanent magnetism (ARM) in samples whereby a peak alternating field of 100 mT was induced with a DC biasing field of 0.04 mT. Remanence was measured in a Molspin magnetometer. Results normalised for the biasing field are termed susceptibility of ARM (χ_ARM_). ARM and χ_ARM_ are sensitive to concentrations of fine-grained (stable single domain, SSD) magnetic particles. SIRM and backfield IRMs were induced in samples using a Molspin Pulse Magnetiser with programmed magnetic field intensities of 800 mT (SIRM) and backfield IRMs of 100 mT (S-RATIO) and 300 mT (HIRM). SIRM reflects the concentration of all remanence-carrying minerals. HIRM shows the relative amount of antiferromagnetic (e.g., haematite) minerals^24^. S-RATIO is the ratio of 100 mT to SIRM and is indicative of mineralogy.

### Geochemistry

Bulk elemental composition of the sediments was determined by XRF (X-ray fluorescence), using a Metorex XMET920^80^. Results were calibrated against a range of materials with certified element concentrations. Using DECONV software^81^, concentration data were normalised for organic matter content, determined by loss on ignition (450 °C). A range of elements were analysed including Al, Br, Ca, Cl, Cr, Cu, Fe, Pb, Mn, Ni, Nb, K, Rb, S, Si, Sr, Ti, Y and Zn. Here, we present results for Pb, Zn and S as indicators of anthropogenic deposition. The detection limits for Pb was 27.0 µg g^−1^ ± 16.5, with a measured maximum of 282.3 µg g^−1^, a minimum of 37.1 µg g^−1^ and a mean of 177.5 µg g^−1^; S was 1.3 mg g^−1^ ± 0.18, with a measured maximum of 31.8 mg g^−1^, a minimum of 3.1 mg g^−1^ and a mean of 15.4 mg g^−1^; and Zn was 30.0 µg g^−1^ ± 22, with a measured maximum of 670.7 µg g^−1^, a minimum of 48.3 µg g^−1^ and a mean of 376.6 µg g^−1^ (n = 47, for DDP core).

### SCP and IAS analysis

Spheroidal carbonaceous particles (SCPs), unambiguous indicators of fossil fuel combustion were extracted in 1 cm intervals and inorganic ash spheres (IAS), produced from high temperature combustion^82^, were purified from selected samples and quantified under a light microscope (× 400) using a known concentration of *Lycopodium* pollen.

### Radiometric dating

Radiometric isotope chronologies were determined by direct gamma assay and dates corrected using the 1963 depth determined from ^137^Cs activity^83^. Down-core geochemical, SCP and magnetic concentrations were converted to flux data using sediment accumulation rates (g cm^−2^ y^−1^) derived from the ^210^Pb chronology^84^.

### SEM analysis

SEM–EDS data for SCPs and IAS were acquired manually using a Hitachi S-3200N scanning electron microscope and an Oxford Link INCA 300 EDS was used to determine elemental composition at 20 kV in ‘spot’ source mode focused on each individual particle for 60 s. IAS were analysed under low vacuum conditions in back scatter electron (BSE) mode. SCP samples were coated with 10 nm of Au and Pd and analysed under high vacuum conditions.

### Characterisation of PM_10_ from an AQMS

Tapered Element Oscillating Microbalance (Rupprech and Patashnick Co Inc) filters, archived from an air quality monitoring station (NGR: SJ 43887, 83600) from Speke, south Merseyside, were wetted with 50 µl 70% ethanol and particles were removed using 5 ml deionised water and filtered through a 40 µm sieve (BD Falcon 352340). Particles were suspended in phosphate buffered saline (PBS) (0.01 mM Na_2_PO_4−_⋅7H_2_O; 3 mM KCl; 140 mM NaCl; pH 7.4), analysed and sorted using a Fluorescence Activated Cell Sorter (BD FACS Aria II). Calibration microspheres were used to determine two sorting gates based on particle size (< 2.5 µm and 2.5–15 µm) and 1,000,000 events were analysed for each gate. Sorted particles were washed five times with dH_2_O and harvested by centrifugation at 1500 rpm for 20 min. PM were transferred to a carbon filter paper and images were acquired via SEM–EDS as described above. High-throughput automated PM analysis was also performed using a Tescan Mira FEG SEM with Aztec Energy Automatic (version 3.1) EDS system, and Aztec Feature particle analysis (Oxford Instruments).

## Supplementary Information

Supplementary Information.

## Data Availability

The datasets used and/or analysed during the current study available from the corresponding author on reasonable request.

## References

[1] World Health Organisation. *Air Pollution the Invisible Killer*. https://www.who.int/health-topics/air-pollution (Accessed 20 May 2019).

[2] Schraufnagel DE (2019). Air pollution and noncommunicable diseases: A review by the Forum of International Respiratory Societies' Environmental Committee, Part 2: Air pollution and organ systems. Chest.

[3] Kelly FJ, Fussell JC (2015). Air pollution and public health: Emerging hazards and improved understanding of risk. Environ. Geochem. Health.

[4] Yue H, He C, Huang Q, Yin D, Bryan BA (2020). Stronger policy required to substantially reduce deaths from PM2.5 pollution in China. Nat. Commun..

[5] Brugha R, Grigg J (2014). Urban air pollution and respiratory infections. Paediatr. Respir. Rev..

[6] Andersen ZJ (2011). Chronic obstructive pulmonary disease and long-term exposure to traffic-related air pollution. Am. J. Respir. Crit. Care Med..

[7] Beelen R (2014). Long-term exposure to air pollution and cardiovascular mortality: An analysis of 22 European cohorts. Epidemiology.

[8] Pope CA (2015). Relationships between fine particulate air pollution, cardiometabolic disorders, and cardiovascular mortality. Circ. Res..

[9] Maher BA (2016). Magnetite pollution nanoparticles in the human brain. Proc. Natl. Acad. Sci. U.S.A..

[10] Anenberg SC, Horowitz LW, Tong DQ, West JJ (2010). An estimate of the global burden of anthropogenic ozone and fine particulate matter on premature human mortality using atmospheric modeling. Environ. Health Perspect..

[11] Wong IC, Ng YK, Lui VW (2014). Cancers of the lung, head and neck on the rise: Perspectives on the genotoxicity of air pollution. Chin. J. Cancer.

[12] Cory-Slechta DA, Sobolewski M (2023). Neurotoxic effects of air pollution: An urgent public health concern. Nat. Rev. Neurosci..

[13] Gong T (2017). Perinatal exposure to traffic-related air pollution and autism spectrum disorders. Environ. Health Perspect..

[14] Bongaerts E (2022). Maternal exposure to ambient black carbon particles and their presence in maternal and fetal circulation and organs: An analysis of two independent population-based observational studies. Lancet Planet Health.

[15] Landrigan PJ (2017). The Lancet Commission on pollution and health. The Lancet..

[16] Lo WC, Hu TH, Hwang JS (2023). Lifetime exposure to PM(2.5) air pollution and disability-adjusted life years due to cardiopulmonary disease: A modeling study based on nationwide longitudinal data. Sci. Total Environ..

[17] Swanton C (2022). LBA1—Mechanism of Action and an Actionable Inflammatory Axis for Air Pollution Induced Non-small Cell Lung Cancer: Towards Molecular Cancer Prevention.

[18] Li X, Jin L, Kan H (2019). Air pollution: A global problem needs local fixes. Nature.

[19] Liang L, Gong P (2020). Urban and air pollution: A multi-city study of long-term effects of urban landscape patterns on air quality trends. Sci. Rep..

[20] Moreno T, Gibbons W, Jones T, Richards R (2004). Geochemical and size variations in inhalable UK airborne particles: The limitations of mass measurements. J. Geol. Soc. Lond..

[21] Hansell A (2016). Historic air pollution exposure and long-term mortality risks in England and Wales: Prospective longitudinal cohort study. Thorax.

[22] Sainsbury P, Hussey R, Ashton J, Andews B (1996). Industrial atmospheric pollution, historical land use patterns and mortality. J. Public Health Med..

[23] Power AL (2018). Monitoring impacts of urbanisation and industrialisation on air quality in the anthropocene using urban pond sediments. Front. Earth Sci..

[24] Walden J, Oldfield F, Smith J (1999). Environmental Magnetism: A Practical Guide. Technical Guide, No. 6.

[25] Robinson SG (1986). The late Pleistocene palaeoclimatic record of North Atlantic deep-sea sediments revealed by mineral-magnetic measurements. Phys. Earth Planet. Interior.

[26] King JW, Channel JET (1991). Sedimentary magnetism, environmental magnetism and magnetostratigraphy. Rev. Geophys..

[27] Maher BA, Thompson R (1999). Climates, Environments and Magnetism.

[28] Dearing JA, Hay KL, Baban SMJ, Huddleston AS, Wellington EMH, Loveland PJ (1996). Magnetic susceptibility of soil: An evaluation of conflicting theories using a national data set. Geophys. J. Int..

[29] Vachula RS, Ojeda AS, Henderson ED, Inoue J (2023). The DiSCPersal model: A simple model for the small-scale atmospheric transport of spheroidal carbonaceous particles (SCPs). Chemosphere..

[30] Matzka J, Maher BA, Moore C (2008). Spatial variation in vehicle-derived metal pollution identified by magnetic and elemental analysis of roadside tree leaves. Atmos. Environ..

[31] Yang T, Liu Q, Li H, Zeng Q, Chan L (2010). Anthropogenic magnetic particles and heavy metals in the road dust: Magnetic identification and its implications. Atmos. Environ..

[32] Hunt A (1986). The application of mineral magnetic methods to atmospheric aerosol discrimination. Phys. Earth Planet. Interior.

[33] Oldfield F (2014). Can the magnetic signatures from inorganic fly ash be used to mark the onset of the Anthropocene?. Anthropocene Rev..

[34] Oldfield F (2015). The magnetic record of inorganic fly ash deposition in lake sediments and ombrotrophic peats. The Holocene.

[35] Mitchell R, Maher BA (2009). Evaluation and application of biomagnetic monitoring of traffic-derived particulate pollution. Atmos. Environ..

[36] Matzka J, Maher BA (1999). Magnetic biomonitoring of roadside tree leaves: Identification of spatial and temporal variations in vehicle-derived particulates. Atmos. Environ..

[37] Robertson DJ, Taylor KG, Hoon SR (2003). Geochemical and mineral magnetic characterisation of urban sediment particulates, Manchester, UK. Appl. Geochem..

[38] Blaha U, Sapkota B, Appel E, Stanjek H, Rösler W (2008). Micro-scale grain-size analysis and magnetic properties of coal-fired power plant fly ash and its relevance for environmental magnetic pollution studies. Atmos. Environ..

[39] Magiera T, Gołuchowska B, Jabłońska M (2012). Technogenic magnetic particles in alkaline dusts from power and cement plants. Water Air Soil Pollut..

[40] Rose NL (1996). Inorganic fly-ash spheres as pollution tracers. Environ. Pollut..

[41] Jones S, Richardson N, Bennett M, Hoon SR (2015). The application of magnetic measurements for the characterization of atmospheric particulate pollution within the airport environment. Sci. Total Environ..

[42] Sheikh HA, Maher BA, Karloukovski V, Lampronti GI, Harrison RJ (2022). Biomagnetic characterization of air pollution particulates in Lahore, Pakistan. Geochem. Geophys. Geosyst..

[43] Xiao H (2022). Prediction of heavy metals in airborne fine particulate matter using magnetic parameters by machine learning from a metropolitan city in China. Atmos. Pollut. Res..

[44] Maity R (2022). Mineral magnetic and geochemical characterization of the dust and soils around Mejia Thermal Power Plant, West Bengal: Implications to source apportionment. J. Earth Syst. Sci..

[45] Jones AD (1969). Publicity & Information services Department.

[46] Barker TC, Harris JR (1959). A Merseyside town in the Industrial Revolution St Helens 1750–1900.

[47] Warren K (1980). Chemical Foundations: The Alkali Industry in Britain to 1926.

[48] Rintoul, G. *Chemical Manufacture in Runcorn and Weston 1800–1930* (1984).

[49] Dingle AE (1982). ‘The monster nuisance of all’: Landowners, alkali manufacturers and air pollution 1828–64. Econ. Hist. Rev..

[50] Carter, D. H. *ICI Magazine* 207 (1964).

[51] Campbell WA (1971). The Chemical Industry.

[52] Halton Borough Council. *Halton Science Report* (2005).

[53] Halton Borough Council. *The Halton Legacy* (1991).

[54] Charlesworth SM, Foster IDL (1999). Sediment budgets and metal fluxes in two contrasting urban lake catchments in Coventry, UK. Appl. Geogr..

[55] Graney JR, Eriksen TM (2004). Metals in pond sediments as archives of anthropogenic activities: A study in response to health concerns. Appl. Geochem..

[56] Fox WM, Johnson MS, Jones SR, Leah RT, Copplestone D (1999). The use of sediment cores from stable and developing salt marshes to reconstruct historical contamination profiles in the Mersey Estuary, UK. Mar. Environ. Res..

[57] Salt J (1968). The motor industry on Merseyside. Geography.

[58] Dockerill B, Sturzaker J (2019). Green belts and urban containment: The Merseyside experience. Plan. Perspect..

[59] Wainwright, S. *Bold Power Station (1955–1986)*. https://www.suttonbeauty.org.uk/suttonhistory/bold_power_station/ (2020).

[60] Hayes E, Charlesworth S, Booth CA (2019). Evolution of air quality policy and management in urban areas. Urban Pollution: Science and Management.

[61] Yang H (2023). Evolution of magnetic particulate matter during its emission process in thermal power plants. Environ. Sci. Nano.

[62] Dale FW (1976). VI—Fiddler's Ferry. Atmos. Environ..

[63] Europe, F. A. W. *Ineos’ Chequered Environmental Track Record in Europe*. https://foodandwaterwatch.org/wp-content/uploads/2021/03/ib_1710_uslet_ineosenvirorecord-web2.pdf (2017).

[64] Hatfield R (2014). Particle size-specific magnetic measurements as a tool for enhancing our understanding of the bulk magnetic properties of sediments. Minerals.

[65] Hunt A, Jones J, Oldfield F (1984). Magnetic measurement and heavy metals in atmospheric particulates of anthropogenic origin. Sci. Total Environ..

[66] Xie RK, Seip HM, Leinum JR, Winje T, Xiao JS (2005). Chemical characterization of individual particles (PM10) from ambient air in Guiyang City, China. Sci. Total Environ..

[67] Casotti Rienda I (2023). PM10 resuspension of road dust in different types of parking lots: Emissions, chemical characterisation and ecotoxicity. Atmosphere.

[68] Liu H (2019). Magnetic signatures of natural and anthropogenic sources of urban dust aerosol. Atmos. Chem. Phys..

[69] Šorša A, Miler M, Gosar M, Halamić J (2018). Follow-up geochemical studies and mineralogical investigations by scanning electron microscopy/energy dispersive spectroscopy (SEM/EDS) of soil samples from the industrial zone of Sisak, Croatia. J. Geochem. Explor..

[70] Zeb B (2022). Variation in coarse particulate matter (PM10) and its characterization at multiple locations in the Semiarid Region. Front. Environ. Sci..

[71] Teran K, Zibret G, Fanetti M (2020). Impact of urbanization and steel mill emissions on elemental composition of street dust and corresponding particle characterization. J. Hazard Mater..

[72] Vlasov D, Kosheleva N, Kasimov N (2021). Spatial distribution and sources of potentially toxic elements in road dust and its PM(10) fraction of Moscow megacity. Sci. Total Environ..

[73] Kasimov NS, Vlasov DV, Kosheleva NE (2020). Enrichment of road dust particles and adjacent environments with metals and metalloids in eastern Moscow. Urban Clim..

[74] Delly JG, McCrone WM (1973). The Particle Atlas: An Encycolpoedia of Techniques Form Small Particle Identification. The Electron Microscopy Atlas.

[75] Chen J, Hoek G (2020). Long-term exposure to PM and all-cause and cause-specific mortality: A systematic review and meta-analysis. Environ. Int..

[76] Pope CA, Dockery DW (2006). Health effects of fine particulate air pollution: Lines that connect. J. Air Waste Manag. Assoc..

[77] Zheng Z (2015). Exposure to fine airborne particulate matters induces hepatic fibrosis in murine models. J. Hepatol..

[78] Tagliabue G (2016). Atmospheric fine particulate matter and breast cancer mortality: A population- based cohort study. Br. Med. J..

[79] Calderon-Garciduenas L (2019). Combustion- and friction-derived magnetic air pollution nanoparticles in human hearts. Environ. Res..

[80] Boyle JF (2000). Rapid elemental analysis of sediment samples by isotope source XRF. J. Paleolimnol..

[81] Boyle JF (1999). Isotope-source, energy-dispersive XRF analysis of geological materials using gas-filled proportional counters: Signal deconvolution using simulated peak shapes. X-Ray Spectrom..

[82] Rose NL (1990). A method for the selective removal of inorganic ash particles from lake sediments. J. Paleolimnol..

[83] Appleby PG (1986). Pb-210 dating by low background gamma-counting. Hydrobiologia.

[84] Norton SA (1992). Stratigraphy of total metals in PIRLA sediment cores. J. Paleolimnol..

