# Supplementary material for: The evolution of atmospheric particulate matter in an urban landscape since the Industrial Revolution
Source: Sci Rep. 2023 Jun 2;13:8964. doi: 10.1038/s41598-023-35679-3 (PMC10238512; doi:10.1038/s41598-023-35679-3)
Supplement: Supplementary file 1 — Supplementary Information. [file 41598_2023_35679_MOESM1_ESM.pdf]

SUPPLEMENTARY MATERIALS

The evolution of atmospheric particulate matter in an urban landscape since the Industrial Revolution

Ann L. Power, Richard K. Tennant, Alex G. Stewart, Christine Gosden, Annie. T Worsley,

Richard Jones and John Love

Table S1. Health and socio-economic indicators for Liverpool and Halton local authority units. Socio-economic and health statistics ranging from 2012-2015 were compiled from Public Health England records (fingertips.phe.org.uk/profile/health-profiles and healthierlives.phe.org.uk). Statistics were compared and ranked (best to worst) according to available data for all District and Unitary Authorities, *County and Unitary Authorities, and **Clinical Commissioning Groups in England. Socio-economic and health indicators that score in the 5th poorest percentile in the National Rank are shaded in light grey. Indicators that score in the 2nd poorest percentile in England are shaded in dark grey.

Socio-economic and Health Indicators		Statistical Indicator			Ranking	
		Liverpool	Halton	England	National Rank Liverpool / England	National Rank Halton / England
Socio-economic	Deprivation[1]	60.5	48.3	20.2	326 / 326	310 /326
	Children in poverty[2]	31.6	24.5	18.6	319 / 325	286 / 325
	Violent crime[3]	17.0	14.4	13.5	260 / 326	219 / 326
	Smoking prevalence[4]	21.7	17.8	18.0	268 / 324	167 / 324
	< 18 years conceptions[5]	32.5	31.5	22.8	288 / 324	283 / 324
	Alcohol related admissions[6]	903.0	805.0	641.0	317 / 326	299 / 326
	Opiate / crack use[7]	1.65	0.84	0.84	314 / 324	221 / 324
Life Expectancy / Deaths	Male Life expectancy (years at birth)	76.4	77.3	79.5	320 / 324	307 / 324
	Female Life expectancy (years at birth)	80.5	80.5	83.2	317 / 324	320 / 324
	Stillbirth / neonatal mortality[7]	0.79	0.69	0.73	225 / 324	164 / 324
	Infant mortality[8]	4.2	2.9	4.0	221 / 326	241 / 326
	Preventable deaths (< 75 years)[6]	280.1	254.0	182.7	323 / 325	314 / 325
Cardiovascular Disease	Cardiovascular mortality[9]	99.5	99.6	75.7	297 / 324	298 / 324
	Heart disease (deaths)[10]	50.7	60.7	41.5	268 / 324	208 / 324
	Stroke (deaths)[10]	19.3	13.8	16.6	258 / 284	218 / 284
Respiratory Disease	Lung disease (deaths)[10]	67.5	50.4	32.6	322 / 323	307 / 323
	COPD prevalence**[17]	2.9	2.6	1.8	200 / 209	184 / 209
	Asthma prevalence**[17]	5.6	6.8	6.0	50 / 209	195 / 209
	Smoking related deaths18	428.3	392.3	274.8	321 / 324	312 / 324
Cancer	Cancer mortality[9]	191.2	187.4	141.5	323 / 324	321 / 324
	Lung cancer deaths[10]	107.2	100.7	59.5	323 / 324	319 / 324
	Breast cancer deaths[10]	20.8	29.5	21.9	92 / 273	259 / 273
	Colorectal cancer deaths[10]	16.5	13.7	12.3	273 / 281	201 / 281
Neurological Disorders	Autism*[11]	1.65	1.25	1.08	139 / 152	112 / 152
	Learning disability all ages*[4]	0.49	0.63	0.44	102 / 152	145 / 152
	Moderate learning disability adults*[12]	4.36	4.67	3.73	121 / 152	135 / 152
	Moderate learning difficulties children*[13]	30.4	30.4	28.6	89 / 148	90 / 148
	Dementia age standardized*[14]	871.0	1035.0	746.0	123 / 152	152 / 152
Other Medical Conditions	TB incidence[15]	8.8	1.9	13.5	224 / 326	29 / 326
	Liver disease deaths[6]	34.3	24.2	17.8	298 / 302	263 / 302
	Obese adults[7]	26.4	33.7	24.0	250 / 326	323 / 326
	Diabetes[16]	6.0	7.6	6.4	110 / 326	293 / 326

1: % population living in 20 % most deprived areas. 2: % of children in low-income families (in receipt of out of work benefits or tax credits where income is < 60 % of median income, for under 16s only). 3: Offences per 1,000 population. 4: % in persons > 18 years. 5: Per 1,000 females (15-17 years). 6: Per 100,000 population. 7: Per 1,000 population. 8: Infant (< 1 year) deaths per 1,000 live births. 9: Age-standardised mortality rate (per 100,000 population < 75 years). 10: Per 100,000 population (< 75 years). 11: % school pupils with diagnosed autism spectrum disorder. 12: Adults 18-64 years receiving long-term support from local authority per 1,000 population. 13: Known to schools per 1,000 pupils. 14: Age-standardised rate of mortality in persons > 65 years with recorded mention of dementia per 100,000 population. 15: Reported cases per 100,000 population. 16: % recorded cases > 17 years registered with a GP. 17: % prevalence all ages. 18: Per 100,000 population > 35 years. 18: %. Spatial health inequalities in England and Wales are presented in Figure S1.

Table S2. Radiometric ^{210}Pb chronology for Daresbury Delph Pond showing depth age and sedimentation rate accuracies.

Depth		Chronology			Sedimentation Rate		
		Date	Age				
cm	g cm^{-2}	AD	y	±	g cm^{-2} y^{-1}	cm y^{-1}	± (%)
0.0	0.00	2005	0	0			
0.5	0.01	2004	1	0	0.020	0.34	18.2
1.0	0.04	2003	2	1	0.020	0.30	18.2
1.5	0.09	2001	4	2	0.020	0.23	18.2
2.0	0.14	1998	7	2	0.022	0.20	18.2
2.5	0.20	1996	9	3	0.025	0.19	18.2
3.0	0.26	1993	12	3	0.025	0.17	18.2
3.5	0.32	1990	15	4	0.019	0.16	18.2
4.0	0.39	1987	18	4	0.018	0.15	18.2
4.5	0.47	1983	22	5	0.021	0.14	18.2
5.0	0.54	1979	26	6	0.020	0.15	18.2
5.5	0.60	1976	29	7	0.018	0.16	22.9
6.0	0.66	1973	32	7	0.017	0.17	25.5
6.5	0.71	1970	35	8	0.017	0.17	27.6
7.0	0.76	1967	38	8	0.017	0.17	28.6
7.5	0.81	1964	41	8	0.016	0.17	28.6
8.0	0.85	1962	43	8	0.017	0.17	28.6
8.5	0.90	1959	46	9	0.019	0.16	28.6
9.0	0.96	1956	49	9	0.019	0.14	28.6
9.5	1.04	1952	53	10	0.018	0.13	28.6
10.0	1.11	1948	57	11	0.017	0.12	28.6
10.5	1.17	1944	61	12	0.017	0.11	28.6
11.0	1.25	1939	66	13	0.016	0.10	28.6
11.5	1.34	1934	71	14	0.016	0.09	28.6
12.0	1.44	1928	77	15	0.016	0.09	28.6
12.5	1.53	1922	83	17	0.016	0.08	28.6

Table S3. Radiometric ^{210}Pb chronology for Oglet Pond showing depth age and sedimentation rate accuracies.

Depth		Chronology Date	Age		Sedimentation Rate		
cm	g cm^{-1}	AD	y	±	g cm^{-2} y^{-1}	cm y^{-1}	± (%)
0.00	0.00	2000	0	0			
0.50	0.05	1999	1	2	0.069	0.58	18.0
1.25	0.14	1998	2	2	0.087	0.75	50.3
1.75	0.20	1997	3	2	0.096	0.75	71.9
2.25	0.26	1997	3	2	0.089	0.67	93.4
2.75	0.33	1996	4	2	0.093	0.67	76.0
3.25	0.41	1995	5	2	0.073	0.50	58.7
3.75	0.48	1994	6	2	0.067	0.50	41.3
4.25	0.55	1993	7	2	0.045	0.50	23.9
4.75	0.60	1992	8	2	0.050	0.67	27.3
5.25	0.65	1991	9	2	0.056	0.67	30.7
5.75	0.70	1991	9	2	0.062	0.50	34.2
6.25	0.75	1990	10	2	0.067	0.50	37.6
6.75	0.81	1988	12	3	0.057	0.33	34.1
7.25	0.86	1987	13	3	0.047	0.33	30.7
7.75	0.92	1985	15	3	0.038	0.33	27.2
8.25	0.98	1984	16	3	0.028	0.33	23.7
8.75	1.03	1982	18	3	0.027	0.33	24.1
9.25	1.08	1981	19	4	0.026	0.29	24.5
9.75	1.12	1979	21	4	0.026	0.22	24.8
10.25	1.17	1977	23	4	0.025	0.18	25.2
10.75	1.24	1973	27	5	0.023	0.15	26.5
11.25	1.31	1970	30	5	0.021	0.14	27.7
11.75	1.39	1966	34	6	0.019	0.15	29.0
12.25	1.46	1963	37	7	0.017	0.15	30.3
12.75	1.55	1960	40	8	0.028	0.17	42.7
13.25	1.64	1957	43	9	0.038	0.17	55.2
13.75	1.72	1954	46	10	0.049	0.22	67.6
14.25	1.81	1951	49	11	0.060	0.29	80.0

Table S4. Radiometric ^{210}Pb chronologies for, Dogs Kennel Clump (DKC1), Speke Hall Lake (SHL1) and Griffin Wood Pond (GWP), showing depth age accuracies.

DKC1					SHL1					GWP				
Depth		Chronology			Depth		Chronology			Depth		Chronology		
		Date	Age				Date	Age				Date	Age	
cm	g cm^{-1}	AD	y	±	cm	g cm^{-2}	AD	y	±	cm	g cm^{-2}	AD	y	±
0	0	2000	0	0	0	0	2001	0	0	0	0	2014	0	0
0.5	0.04	1998	2	2	0.5	0.03	1999	2	1	0.5	0.3	2013	1	0
1.25	0.15	1994	6	2	1.5	0.1	1994	7	2	2.5	1.9	2006	8	1
1.75	0.25	1988	12	2	2.5	0.19	1987	14	2	4.5	3.6	1999	15	2
2.25	0.35	1983	17	2	3.5	0.28	1979	22	2	6.5	5.3	1992	22	3
2.75	0.47	1979	21	3	4.5	0.36	1972	29	3	8.5	7	1984	30	3
3.25	0.58	1974	26	4	5.5	0.46	1963	38	4	10.5	8.8	1974	40	4
3.75	0.68	1972	28	4	6.5	0.55	1955	46	5	12.5	10.6	1963	51	5
4.25	0.79	1970	30	4	7.5	0.65	1949	52	6	14.5	12.4	1953	61	6
4.75	0.9	1969	31	5	8.5	0.76	1942	59	8	16.5	14.2	1945	69	7
5.25	1.02	1967	33	5	9.5	0.88	1932	69	10	18.5	15.9	1937	77	8
5.75	1.15	1965	35	5	10.5	1.01	1923	78	13	20.5	17.8	1926	88	9
6.25	1.28	1963	37	6	11.5	1.15	1912	89	18					
6.75	1.43	1963	37	6	*12.5*	1.30	1902	99						
7.25	1.58	1962	38	7	*14.5*	*1.63*	*1881*	*120*						
7.75	1.73	1962	38	8										
8.25	1.88	1962	38	9										
8.75	2.08	1960	40	10										
9.25	2.28	1959	41	12										
9.75	2.48	1958	42	13										
10.5	2.79	1955	45	15										

Table S5. Characterisation of magnetic grain size using magnetic parameters [1-12]. Reported magnetic domain sizes [4] and [1] are included.

Magnetic grain size	Indicator	Potential source	Diagnostic characteristics
Super paramagnetic (SP) (<0.02 μm) [< 0.05 μm]	χ_{FD} % > 10	Topsoil input	Low SIRM/χ_{LF} ratios; χ_{ARM}/χ_{FD} versus χ_{ARM}/χ_{LF} bi-plot with high >8% χ_{FD}.
Single stable domain (SSD) (0.03 to 0.1 μm) [0.07 to 0.7 μm]	χ_{ARM}/ SIRM > 70 10^{-5} Am^{-1} and χ_{DF} > 2 %	SSD bacterial magnetosomes	χ_{ARM}/χ_{FD} versus χ_{ARM}/χ_{LF} bi-plot, χ_{ARM}/SIRM > 2 10^{-3} mA.
	high SIRM/χ_{LF} and χ_{ARM}/SIRM	Greigite	SIRM/χ_{LF} 50 to 80 k Am^{-1}coinciding with high loss of remanence between -40 mT and -100 mT and low HARD and SOFT.
		Dissolution of fine magnetic grains down-core	Coarsening of the magnetic signal combined with very low χ_{LF} (reduced to zero).
Shift to finer magnetic signal / pseudo single domain (PSD)	Proportionately higher χ_{ARM} values compared to χ_{LF} and SIRM	Increased supply of relatively fine grains	S-RATIO -0.4 to -0.7 highlights SD and / or mix of antiferromagnetic grains. Increases in SIRM/χ_{LF} may highlight a decrease in grain size.
(0.1 to 20 μm) [0.7 to 10 μm]		Pollution particles	Fine-grained magnetite (0.2 to 5 μm) as identified in household dust (PSD MD and SP size fractions). Iron-rich spheres from the high temperature combustion of fossil fuels. Soot from petrol and diesel engines contain mainly 0.1 – 1 μm ferrimagnetic minerals. Combustion signals from aircraft engines are a mix of ferrimagentic and antiferromagnetic particles. Coal combustion produces characteristic fine (< 2 μm) magnetite and haematite spherules (IAS).
Multi domain (MD) (> 20 μm) [>10 μm]	High SIRM/ARM ratios and low χ_{ARM}	Pollution particles	S-RATIO -0.7 to -1.0 demonstrates dominance of soft MD grains, typically relatively coarse IAS (> 2 μm) from coal combustion. Metal-rich brake and tyre abrasion particles from road vehicles and aircrafts display coarse ferrimagentic MD signal. Fe-rich spheres 2- 70 μm derived from traffic emissions.
		Unweathered bedrock	Low SIRM/χ_{LF} quotients (< 10 kAm^{-1}) and low χ_{FD}% (<1).

Table S6. Statistical relationships revealed by Spearman's rank correlation between magnetic susceptibility (χ_{LF}), anhysteretic remanent magnetisation (χ_{ARM}), Pb, S, Zn, saturation isothermal remanence magnetisation (SIRM) and Hard isothermal remanence magnetisation (HIRM) concentrations recorded in sediments of Daresbury Delph Pond. P values indicated by *** <0.0001; **< 0.001 and * < 0.05. n = 47 (except for SCP data n= 24).

	χ_{LF}	χ_{ARM}	S	SCP	Pb	Zn	SIRM	HIRM
χ_{LF}	1.00							
χ_{ARM}	0.878***	1.00						
**S**	-0.507*	-0.537***	1.000					
**SCP**	0.597**	0.695**	0.014	1.000				
**Pb**	-0.272	-0.366*	0.715***	0.221	1.000			
**Zn**	0.256	0.184	0.187	0.694**	0.548***	1.000		
**SIRM**	0.937***	0.940***	-0.455*	0.737***	-0.268	0.362*	1.000	
**HIRM**	0.801***	0.939***	-0.446*	0.622*	-0.329	0.168	0.889***	1.000

Table S7. Mean flux values for temporal phases. Temporal divisions of 25 years represent generational timescales, with pre-industrial means calculated from 1700-1800 sediment, a time of minimal, small-scale industrial activity in the Merseyside region. Mean values for each time interval were normalised for the pre-industrial mean to calculate enrichment factors.

**Temporal phase**	**SIRM (10^{-3} A y^{-1})**	**HIRM (10^{-3} A y^{-1})**	**Pb (μg cm^{-2} y^{-1})**	**Zn (μg cm^{-2} y^{-1})**	**S (mg cm^{-2} y^{-1})**	**SCP (cm^{-2} y^{-1})**
1980-2005	8.104	2.760	2.318	6.790	0.144	222.033
1955-1980	6.456	0.647	3.204	7.965	0.310	368.304
1930-1955	5.004	0.481	3.699	9.062	0.255	194.155
1905-1930	4.317	0.297	3.962	9.651	0.343	204.824
1880-1905	2.954	0.161	3.736	7.352	0.310	107.955
1855-1880	2.688	0.250	6.321	11.265	0.655	72.240
1830-1855	1.184	0.127	2.956	3.290	0.258	32.259
1800-1830	2.340	0.242	2.230	2.240	0.295	28.132
Pre-industrial (1700-1800)	2.264	0.334	1.627	1.885	0.221	27.996

Table S8. Generational changes in pollution proxies. Mean SIRM, HIRM, Pb, Zn, S and SCP flux enrichment factors (anthropogenic-to-natural ratios)[13], for 25-year temporal divisions, representing a human generational timescale, normalised for mean pre-industrial (1700-1800) flux (SI Table S6) for Daresbury Delph Pond. Maximum enrichment factors are highlighted. The highest Pb, Zn and S enrichment was experienced between 1855-1880. A century later (1955-1980), maximum SCP enrichment occurs (13.155 times higher than pre-industrial values). The highest SIRM and HIRM enhancements, proxies for overall PM pollution and relatively fine IAS particulates, respectively, were experienced between 1980-2005. This suggest that populations born in Merseyside post-1980 have experienced relatively lower levels of Pb, Zn and S, but enriched levels of fine PM and combustion-derived particulates (SCPs) during their lifetimes compared to previous generations.

	**Enrichment factor - relative to pre-industrial values**					
**Temporal phase**	**SIRM**	**HIRM**	**Pb**	**Zn**	**S**	**SCP**
1980-2005	2.750	6.335	1.093	2.785	0.505	5.916
1955-1980	2.626	1.788	1.781	3.836	1.304	12.068
1930-1955	2.139	1.373	2.169	4.607	1.122	6.393
1905-1930	1.566	0.735	1.959	4.145	1.262	6.079
1880-1905	0.949	0.358	1.693	2.841	1.053	2.753
1855-1880	1.223	0.733	3.972	6.152	3.084	2.547
1830-1855	0.637	0.458	2.171	2.104	1.417	1.408
1800-1830	0.907	0.609	1.163	1.026	1.113	0.905

Table S9. Statistical relationships revealed by Spearman's rank correlation between post-1960 sulphur concentrations recorded in the sediments of Speke Hall Lake (SHL1) and Daresbury Delph Pond (DDP) with available long-term monitored black smoke and sulphur dioxide concentrations (regional means). P values indicated by *** <0.0001; **< 0.001 and * < 0.05.

**Sulphur concentrations recorded in urban archive**	**Monitored black smoke data (R value)**	**Monitored sulphur dioxide data (R value)**
SHL1 (n=10)	0.879*	0.915**
DDP (n=14)	0.932***	0.960 ***

Table S10. Statistical relationships revealed by Spearman's rank correlation between Pb, S, Zn, Saturation Isothermal Remanence magnetisation (SIRM) and Hard Isothermal Remanence magnetisation (HIRM) flux values recorded in sediments of Oglet Pond, with transport data from Liverpool John Lennon International Airport (obtained from the Civil Aviation Authority http://www.caa.co.uk.). P values indicated by *** <0.0001; **< 0.001 and * < 0.05. n = 22.

	**Terminal passengers (R value)**	**Air transport movements (R value)**
**Pb flux**	0.494	0.769 ***
**S flux**	0.383	0.752***
**Zn flux**	0.442*	0.724**
**SIRM flux**	0.382	0.733**
**HIRM flux**	0.533*	0.692**

Table S11. SEM-EDS analysis of atmospheric filters.

Background elemental contributions (weight %) from a clean carbon filter paper; carbon filter with fibres from a clean TEOM filter; and clean TEOM filter (glass fibre and Teflon).

Potential background elemental contributions from the TEOM filter were analysed via EDS analysis of a clean filter. This revealed the presence of fluorine (41.97 %) from the Teflon (polytetrafluoroethylene) with lesser amounts of Na, Si, K and Zn. Therefore, to reduce this background 'noise', pollution particles were washed from the TEOM filter and transferred onto a carbon filter comprised of C (84.09 %) and O (15.91 %). Since fibres from the TEOM filter may have been dislodged during sonication, we also investigated the background contribution from the carbon filter with fibres from a clean TEOM filter. Contributions of Na, Si, K, Br and Ba were detected, with a combined total of 4.31 %.

**Material**	**Element (wt %)**									
	**C**	**O**	**F**	**Na**	**Al**	**Si**	**K**	**Zn**	**Br**	**Ba**
Clean carbon filter	84.09	15.91	-	-	-	-	-	-	-	-
Carbon filter with fibres from clean TEOM	65.11	30.57	-	1.05	-	1.94	0.02	-	0.98	0.32
Clean TEOM filter	12.56	19.94	41.97	2.53	0.74	13.92	1.38	2.87	1.16	2.93

Table S12. Elemental chemistry, size and morphology properties for PM from TEOM filter. Elemental weight (Wt%): the concentration of an element in weight percentage; area: area of the particle; aspect ratio: longest divided by shortest particle diameter (symmetrical features such as cubes of spheres have an aspect ratio close to 1); breadth: shortest particle diameter; equivalent circle diameter (ECD): the diameter of a circle that has the same area as the particle, regardless of shape.

ECD is used as a primary function of size in this instance as the FC method used spheres to calibrate size. The behaviour of particles in air is more relative to ECD than length; length: longest particle diameter; perimeter: distance of the outer boarder of the particle; shape: indicates the shape of the feature (a circle has a value of 1, whereas elongated and irregular particles have larger values).

A high-throughput, automated SEM particle analysis was applied to PM from the TEOM filter that had been sorted into < 2.5 μm by FC. A total of 3679 particles were analysed for morphological features, size and chemistry (via EDS) to compliment manual characterisation of PM using SEM-EDS. The majority of particles detected had an ECD (equivalent circle diameter) < 5 μm highlighting the use of FC to concentrate particles within the PM_{fine} size fraction from bulk PM_{10} samples. The breadth of particles ranged from 0.05 μm to 43.21 μm. Coarse PM (> 20 μm) are likely to represent particles clumped together during transfer onto filter papers for SEM analysis.

From the automated SEM data coarse (ECD > 2.5 μm) and fine (ECD < 2.5 μm) particles were separated to assess the chemical composition and morphology of particles. Smaller particles were detected however were below the BSE image threshold. Future analysis could incorporate these particles by adjusting detection threshold values. Particles were then classified based on their dominating chemistry.

Element (wt %)	Count	Minimum	Maximum	Mean	St Dev
B	3	64.16	90.51	77.97	13.23
O	3293	11.86	100	67.5	12.11
F	4	13.36	17.50	15.31	1.92
Na	860	0.66	21.84	6.74	3.94
Mg	459	0.41	28.15	5.50	4.64
Al	1832	0.6	41.06	7.78	4.38
Si	2562	0.89	100.00	16.19	8.84
P	47	0.99	18.44	3.91	3.38
S	309	0.64	100.00	8.98	21.63
Cl	114	0.43	45.93	4.29	6.71
K	749	0.59	28.46	3.24	2.38
Ca	146	0.53	25.20	4.93	4.59
Ti	102	0.86	54.75	11.20	10.61
Cr	27	1.45	13.83	5.82	3.56
Mn	7	1.82	4.58	2.94	1.11
Fe	1670	0.72	100.00	21.57	19.03
Ni	7	2.19	18.13	8.95	5.50
Cu	27	1.26	50.65	6.45	9.19
Zn	11	2.45 (10^{-3})	16.81	5.24	6.04
Br	41	1.02	13.95	6.36	3.18
Zr	6	3.90	39.36	21.25	15.35
Mo	27	2.31	100.00	9.83	18.16
Tc	3	19.52	29.24	23.38	5.16
Ag	4	9.8	37.04	24.21	11.77
Cd	1	2.36	2.36	2.36	0.00
In	1	3.07	3.07	3.07	0.00
Sn	4	5.50	45.13	19.20	18.45
Sb	3	2.60	24.82	10.30	12.58
Ba	5	1.41	9.32	4.93	3.12
Ce	1	5.64	5.64	5.64	0.00

Ta	2	0.00	0.03	0.01	0.02
W	1	51.71	51.71	51.71	0.00
Os	1	0.00	0.00	0.00	0.00
Tl	1	11.54	11.54	11.54	0.00
Pb	3	8.25	20.84	12.47	7.25
Bi	3	0.00	46.57	28.61	25.04
Area sq µm	3679	2.87 (10^{-3})	2924.72	19.01	82.39
Aspect ratio	3679	1.05	13.71	1.40	0.45
Breadth µm	3679	0.05	43.21	3.51	2.55
ECD µm	3679	0.06	61.02	3.84	3.08
Length µm	3679	0.08	127.26	5.00	5.35
Perimeter µm	3679	0.19	543.55	13.25	16.74
Shape	3679	0.93	13.66	1.14	0.54

Supporting Figures

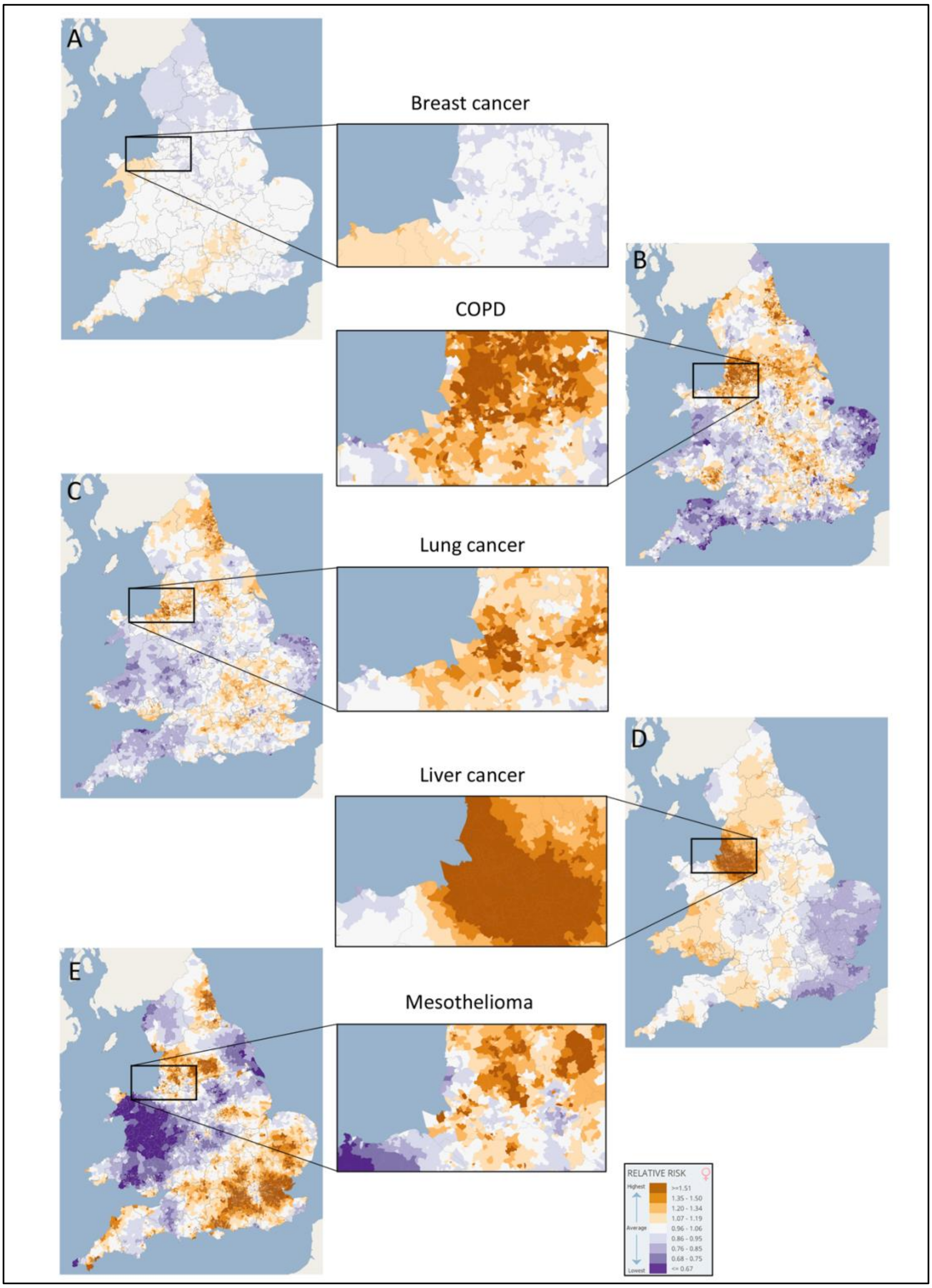

Figure S1. Relative risk of disease at small scale area level (ward) over a 25-year period (1985-2009) in England and Wales. High risk categories (orange) and low risk categories (blue) are shown relate to the average for England and Wales. Risk (age and deprivation adjusted incidence rates) in females for: A: breast cancer, B: COPD (chronic obstructive pulmonary disease), C: lung cancer, D: liver cancer and E: mesothelioma (maps available at www.envhealthatlas.co.uk). The prevalence of COPD, lung cancer, liver cancer and mesothelioma in the NW of England is shown, compared to, for example

limited geographical variation in breast cancers. Relative risk is a comparison of the risk of an area (census ward) relative to the average risk of a health condition in England and Wales (average risk for England and Wales =1.)

Relative risks of brain and breast cancers for Merseyside and Halton are in line with the national average (0.96 – 1.06) with higher than average risk (1.07-1.19) observed in south Sefton and Wirral for brain cancer and the ward of Church in Liverpool (1.07-1.19) for breast cancer. Skin cancer risks are also elevated (1.35-1.50) in serval wards in Liverpool. High relative risks are observed in west Halton and St Helens for kidney disease. Lung cancer, COPD and mesothelioma risks are, however notably elevated above national averages, with maximum relative risks (> 1.51) in wards throughout Halton, Liverpool, Wirral and Knowsley, demonstrating the prevalence of respiratory disease in the region. All wards in Halton, and the majority of wards in Merseyside exhibit maximum relative risk values for liver cancer. Liver cancer incidence exhibits a clear spatial trend of maximum risk concentrated in wards in Merseyside, Halton, Warrington, Lancashire and Cheshire, with a steady reduction with increased distance in all directions, potentially indicating and environmental risk factor.

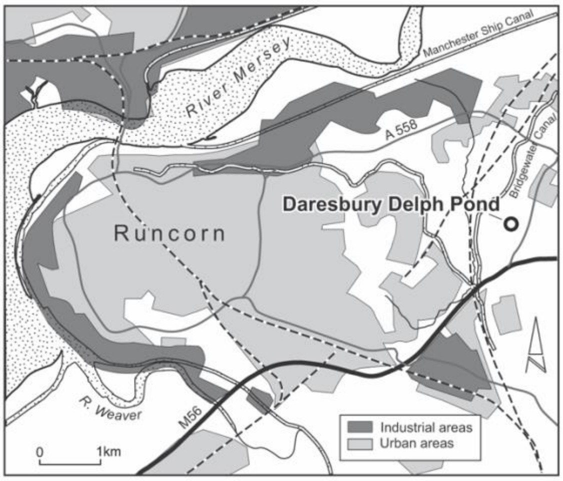

Figure S2. Location of Daresbury Delph Pond (DDP) within the urban landscape.

A: Location map of Daresbury Delph Pond National Grid Reference: SJ 357366, 381975.

Daresbury Delph Pond (DDP) is a small (~768 m^2 surface area, ~2 m maximum water depth) humanmade pond located within the heavily industrialised borough of Halton, NW England (National Grid Reference: SJ 357366, 381975), immediately east of Runcorn. The region, known as the birthplace of the chemical industry, has been dominated by chemical production since the early 19th century and remains a major centre for the production of inorganic and organic chemicals with local industries at Runcorn (5 km east), Weston (6.4 km east), Widnes (7.2 km northeast) and south Liverpool (13 km northwest). Nearby Fiddlers Ferry power station in Warrington (5 km northeast) has been operational since 1973 and surrounding industrial sites include petrochemical plants at plants at Stanlow (15 km southeast) and Ellesmere Port (17 km southeast) and industrial docklands at Bootle (26 km west). The catchment of the pond is defined by highly vegetated steep margins. With a pond-to-catchment ratio of 1:1.4, the primary inorganic contribution to the pond is highly likely to be atmospherically derived. The pond (water surface) is situated at 55 m AOD (Above Ordnance Datum) and local bedrock is comprised of Sherwood Sandstone Group with over lying silts and mudstones from the Merica Mudstone Group and superficial Devensian till deposits. The pond was formed from a marl pit dug pre-mid 19th century out of underlying Bollin mudstone (aka lower Keuper marl). Bollin mudstone is comprised of interlaminated gypsiferous ($CaSO_4$) and anhydritic ($CaSO_4$) mudstone and dolomitic siltstone ($CaMg(CO_3)_2$) with minor amounts of albite ($NaAlSi_3O_8$) and potassium-feldspar ($KAlSi_3O_8$). The examination of maps spanning from the present day to tithe publications (1844) provides confidence in the longevity and minimal disturbance of DDP and its surrounding catchment. Consultation with the landowner and ecologists from the local borough council, at time of core extraction, revealed that there has been no recorded work (such as dredging) performed at the pond.

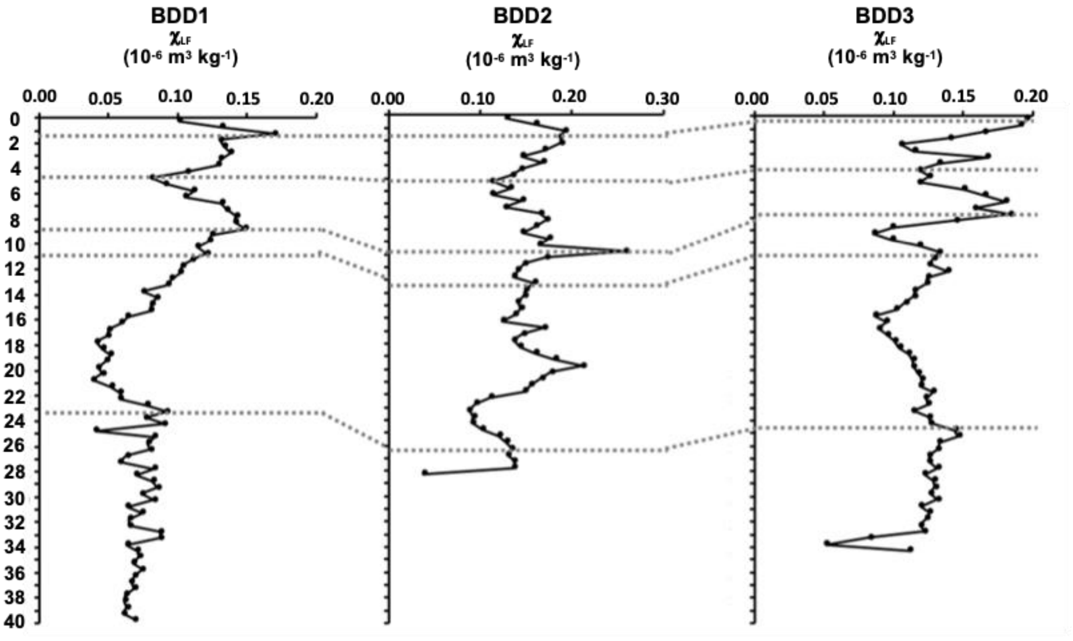

Figure S3. Intra-site visual correlations of magnetic concentration profiles (χ_{LF}) of three cores (BDD1, BDD2 and BDD3) collected from Daresbury Delph Pond, demonstrating the reproducibility and reliability of the sediment record.

Well-matched intra-site magnetic concentration profiles (χ_{LF}) from three DDP cores highlights the reproducibility and reliability of the sediment record. Small ponds can experience periodic drying, which would disturb the accumulation of sediment along the pond basin. 'Noisy' metal and magnetic profiles would indicate such sporadic sediment accumulation events; however, the geomagnetic profiles obtained from DDP do not demonstrate a disrupted signal.

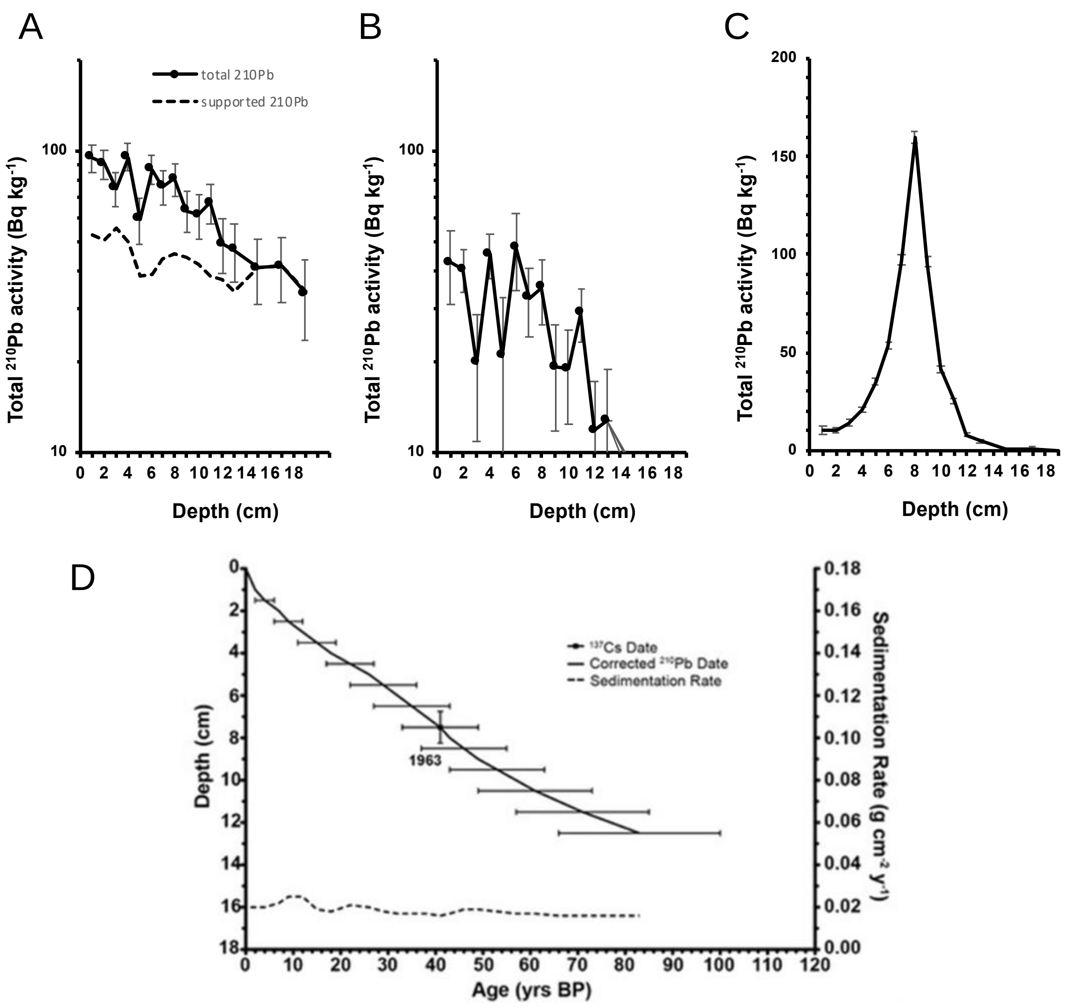

Figure S4. Radiometric chronology for Daresbury Delph Pond.
A and B: Total ^{210}Pb activity reaches equilibrium with that of the supporting ^{226}Ra (equal to supported ^{210}Pb) at a depth of around 14.75 cm. Unsupported ^{210}Pb activity declines irregularly with depth. C: ^{137}Cs activity has a well-resolved peak between 7-8.5 cm that almost certainly records the 1963 fallout maximum from the atmospheric testing of nuclear weapons. D: The CRS (constant rate of supply) dating model was used to calculate ^{210}Pb dates, which were corrected using the 1963 ^{137}Cs depth as a reference. Dates below the ^{210}Pb dating horizon of 12.5 cm were extrapolated back to the start of the sediment record via polynomial trend lines of age versus depth plots. Extrapolated dates and dry bulk density data were used to calculate sediment accumulation rates outside of the CRS model.

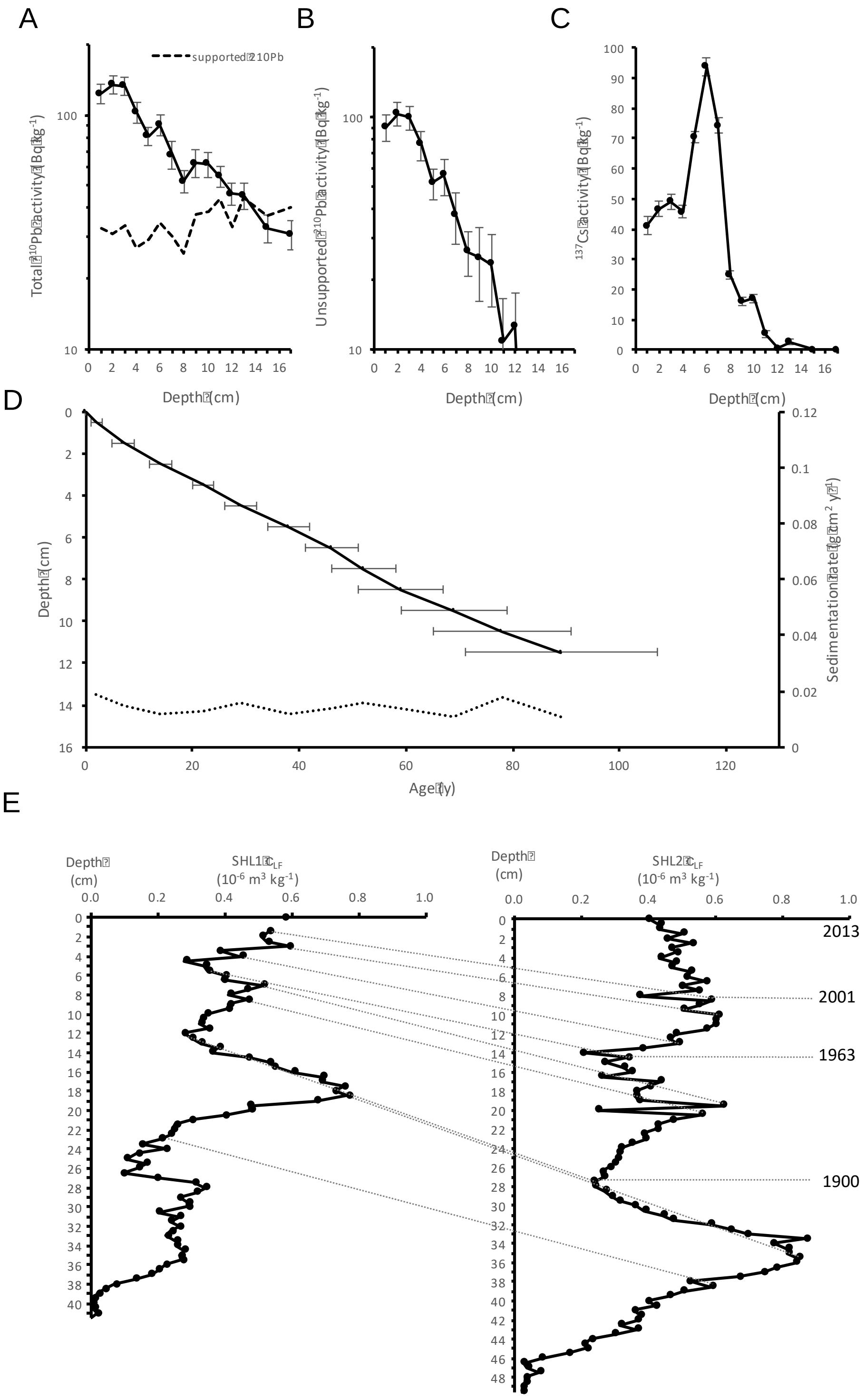

Figure S5: Sediment chronology for Speke Hall Lake (SHL).
A and B: Total ^{210}Pb activity reaches equilibrium with the supported ^{210}Pb at approximately 12 cm. Unsupported ^{210}Pb activity has a maximum value between 1-3 cm and then declines exponentially with depth. C: The ^{137}Cs activity demonstrates a well resolved peak ~5-6 cm that records the 1963 fallout maximum from the atmospheric testing of nuclear weapons, and is in good agreement with the ^{210}Pb dates. D: A high-resolution chronology post-1900 is reconstructed with relatively uniform accumulation sedimentation rate is observed during the 20th century. E: Intra-site core correlations using magnetic susceptibility profiles for SHL1 core collected in 2001 and SHL2 core collected in 2016.

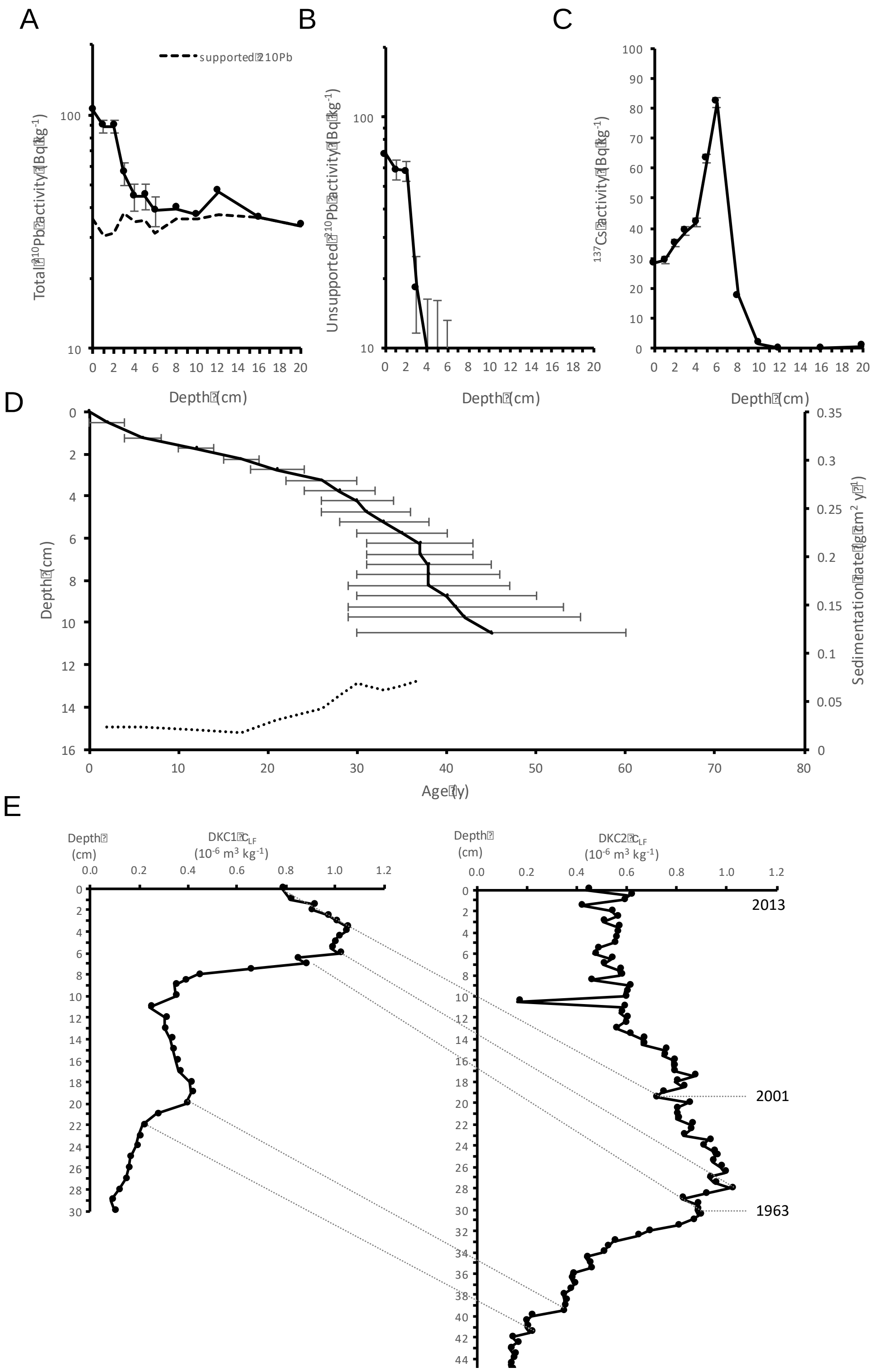

Figure S6: Sediment chronology for Dogs Kennel Clump.

A: Total ^{210}Pb activity is higher than supported ^{210}Pb in the top part of the core only (0-4.25 cm). B: Unsupported ^{210}Pb activity in this surficial zone declines irregularly with depth which is characterised by low density sediment. There is a progressive increase in density with depth to 12.5 cm and an underlying zone of higher compaction. C: The well resolved ^{137}Cs peak very probably reflects the maximum fallout form atmospheric nuclear weapons testing. D: In spite of the disequilibrium in the ^{210}Pb record, CRS modelled dates are consistent with the ^{137}Cs reference point. E: Intra-site core correlations using magnetic susceptibility profiles for DKC1 core collected in 2001 and DKC2 core collected in 2016.

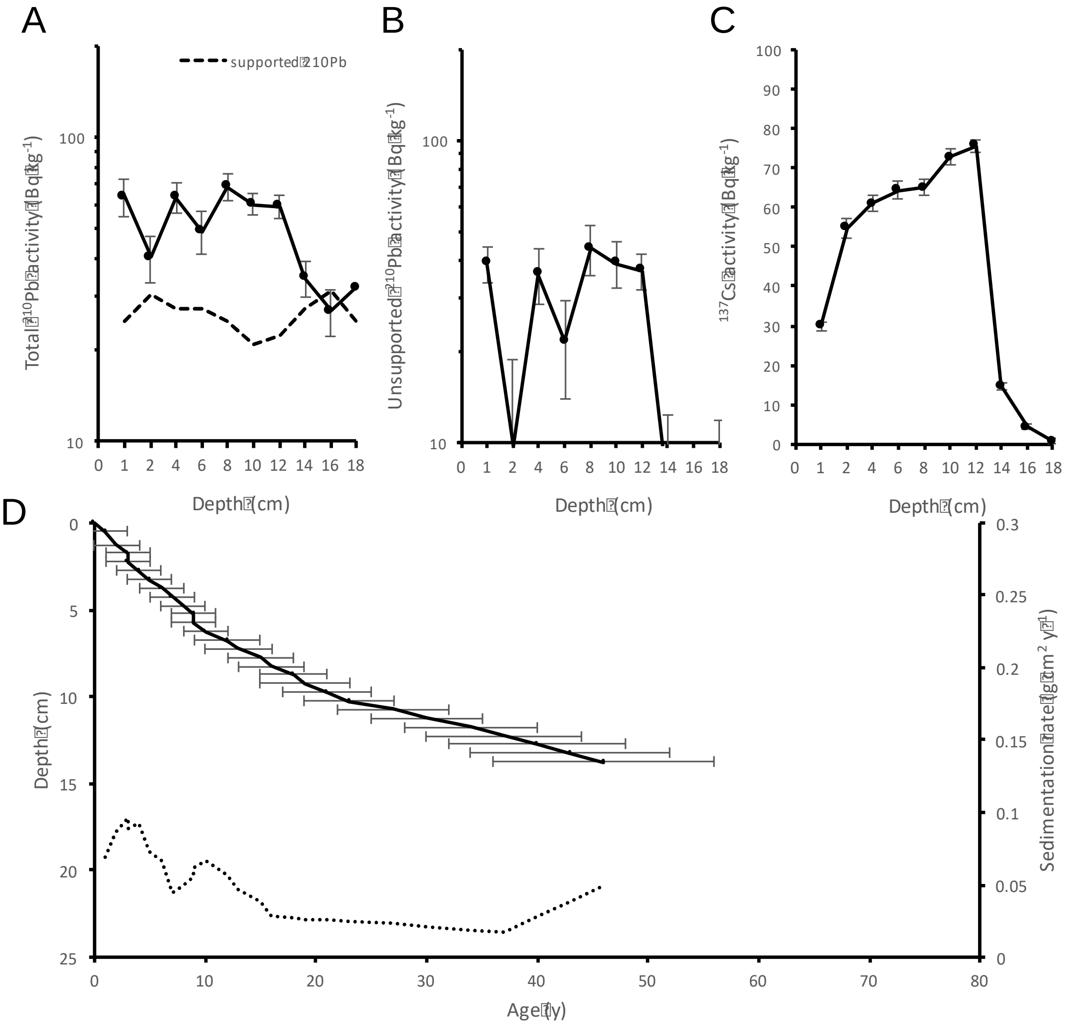

Figure S7: Sediment chronology for Oglet Pond (OG).
A and B: An irregular ^{210}Pb record with no clear equilibrium depth and relatively consistent unsupported ^{210}Pb concentrations are observed in the top 12 cm of the core. Below this, concentrations fall abruptly, with highly compacted sediments in this deeper section. C: The peak ^{137}Cs concentration at 12.25 cm represents maximum fallout in the early 1960s. D: The CRS modelled ^{210}Pb dates places 1963 (at 10.5 cm) in relatively good agreement with the ^{137}Cs dates. Corrected ^{210}Pb dates using the ^{137}Cs reference show a period of rapid sediment accumulation in the 1950s and in the 1990s, with a relatively uniform accumulation of sediment between these events. A high-resolution post-1950 chronology is obtained. Due to the small size of OG pond, multiple cores could not be successfully retrieved without causing sediment disturbance to the sediment basin.

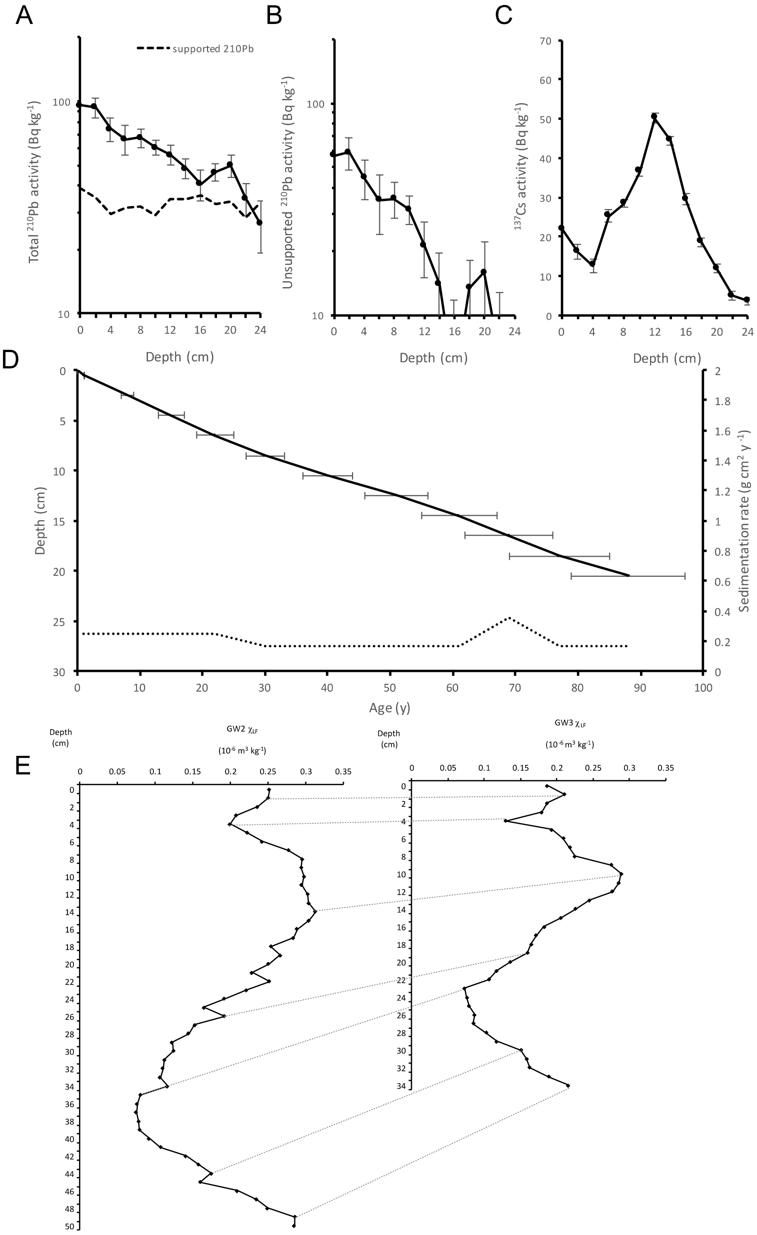

Figure S8: Sediment chronology for Griffin Wood Pond.

A: Total ^{210}Pb activity reaches equilibrium with the supported ^{210}Pb at ~23 cm. B: Maximum unsupported concentrations < 60 Bq kg^{-1} indicate the ^{210}Pb dating horizon is unlikely to be more than 3 half-lives (60-70 years). C: A relatively defined peak at ~12 cm is very likely to represent the 1963 fallout maximum from atmospheric testing of nuclear weapons. D: ^{210}Pb dates calculated using the CRS model places the 1963 in good agreement with the ^{137}Cs record. Slightly higher sedimentation rates are observed post-1990, with relatively uniform sedimentation rates observed from the md-20th century to 1990. An episode of brief rapid sediment accumulation is observed in the 1940s. Sedimentation rates prior to this are uncertain. E: Intra-site core correlations were determined using magnetic susceptibility profiles from multiple extracted cores (GWP2 and GWP3).

Additional References

1. Walden, J.; Oldfield, F.; Smith, J., *Environmental Magnetism: a practical guide. Technical Guide, No. 6*. Quaternary Research Association: London, 1999.
2. Thompson, R.; Thompso, Environmental applications of magnetic measurements. *Science* **1980,** *207*, 481-486.
3. Oldfield, F., Toward the discrimination of fine-grained ferrimagnets by magnetic measurements in lake and near-shore marine sediments. *J. Geophys. Res.* **1994,** *99*, 904-9050.
4. Hatfield, R., Particle Size-Specific Magnetic Measurements as a Tool for Enhancing Our Understanding of the Bulk Magnetic Properties of Sediments. *Minerals* **2014,** *4* (4), 758-787.
5. Oldfield, F., Sources of fine-grained magnetic minerals in sediments: a problem revisited. *The Holocene* **2007,** *17* (8), 1265-1271.
6. Hunt, A., The application of mineral magnetic methods to atmospheric aerosol discrimination. *Physics of the Earth and Planetary Interior* **1986,** *42*, 10-21.
7. Thompson, R.; Oldfield, F., *Environmental Magnetism*. George Allen & Unwin: London, 1986.
8. Maher, B. A.; Thompson, R., *Climates, Environments and Magnetism*. Cambridge University Press: Cambridge, 1999.
9. Górka-Kostrubiec, B.; Jeleńska, M.; Król, E., Magnetic signature of indoor air pollution: Household dust study. *Acta Geophysica* **2014,** *62* (6), 1-26.
10. Jones, S.; Richardson, N.; Bennett, M.; Hoon, S. R., The application of magnetic measurements for the characterization of atmospheric particulate pollution within the airport environment. *The Science of the total environment* **2015,** *502* (C), 385-390.
11. Bućko, M. S.; Magiera, T.; Johanson, B.; Petrovský, E.; Pesonen, L. J., Identification of magnetic particulates in road dust accumulated on roadside snow using magnetic, geochemical and micro-morphological analyses. *Environmental pollution* **2011,** *159* (5), 1266-1276.
12. Robertson, D. J.; Taylor, K. G.; Hoon, S. R., Geochemical and mineral magnetic characterisation of urban sediment particulates, Manchester, UK. *Applied Geochemistry* **2003,** *18*, 269-282.
13. Norton, S. A.; Jr, R. W. B.; Binford, M. W.; Kahl, J. S., Stratigraphy of total metals in PIRLA sediment cores. *J Paleolimnol* **1992,** *7*, 191-214.
